# Structural equation modelling the relationship between anti-fungal prophylaxis and *Pseudomonas* bacteremia in ICU patients

**DOI:** 10.1186/s40635-022-00429-8

**Published:** 2022-01-21

**Authors:** James C. Hurley

**Affiliations:** 1 Melbourne Medical School, University of Melbourne, Melbourne, Australia; 2 Division of Internal Medicine, Ballarat Health Services, PO Box 577, Ballarat, VIC 3353 Australia

**Keywords:** Topical antibiotics, Candidemia, Generalized structural equation modelling, Anti-fungal, *Pseudomonas* bacteremia

## Abstract

**Purpose:**

Animal models implicate candida colonization facilitating invasive bacterial infections. The clinical relevance of this microbial interaction remains undefined and difficult to study directly. Observations from studies of anti-septic, antibiotic, anti-fungal, and non-decontamination-based interventions to prevent ICU acquired infection collectively serve as a natural experiment.

**Methods:**

Three candidate generalized structural equation models (GSEM), with *Candida* and *Pseudomonas* colonization as latent variables, were confronted with blood culture and respiratory tract isolate data derived from 464 groups from 279 studies including studies of combined antibiotic and antifungal exposures within selective digestive decontamination (SDD) interventions.

**Results:**

Introducing an interaction term between *Candida* colonization and *Pseudomonas* colonization substantially improved GSEM model fit. Model derived coefficients for singular exposure to anti-septic agents (− 1.23; − 2.1 to − 0.32), amphotericin (− 1.78; − 2.79 to − 0.78) and topical antibiotic prophylaxis (TAP; + 1.02; + 0.11 to + 1.93) versus *Candida* colonization were similar in magnitude but contrary in direction. By contrast, the model-derived coefficients for singular exposure to TAP, as with anti-septic agents, versus *Pseudomonas* colonization were weaker or non-significant. Singular exposure to amphotericin would be predicted to more than halve candidemia and *Pseudomonas* bacteremia incidences versus literature benchmarks for absolute differences of approximately one percentage point or less.

**Conclusion:**

GSEM modelling of published data supports the postulated interaction between Candida and *Pseudomonas* colonization towards promoting bacteremia among ICU patients. This would be difficult to detect without GSEM modelling. The model indicates that anti-fungal agents have greater impact in preventing *Pseudomonas* bacteremia than TAP, which has no impact*.*

**Supplementary Information:**

The online version contains supplementary material available at 10.1186/s40635-022-00429-8.

## Take home message

Structural equation modelling of the ICU infection prevention data from 279 studies supports the postulated influence of prophylaxis using anti-fungal agents in preventing *Pseudomonas* bacteremia. The model implicates that anti-fungal agents have greater impact in preventing bacteremia versus antibiotics, which have no impact.

## Introduction

**Fig. 1:**
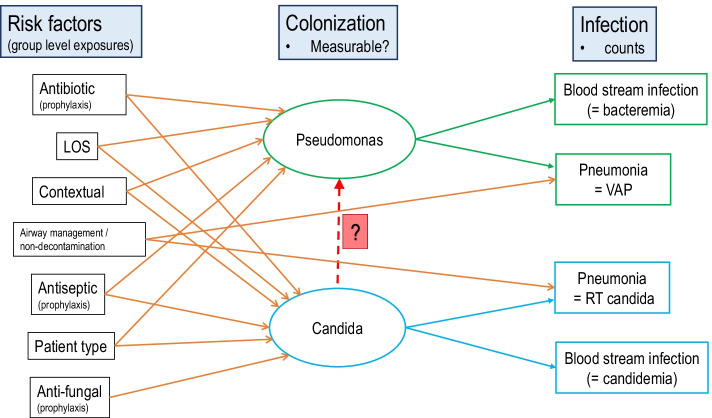
Theoretical model of clinical factors bearing on the interaction between *Pseudomonas* and candida colonization towards causing blood stream and other infections. ‘contextual’ refers to the contextual effect within each ICU setting. The blue boxes label the elements required to address the central research question here depicted by the vertical arrow labelled ‘?’. This research question would not be easily addressed within a single center study

Animal models implicate candida colonization facilitating invasive bacterial infections [1, 2]. Which of over 600 catalogued immunological, biochemical, metabolic and mechanical processes might underlie this interaction between *Pseudomonas aeruginosa* and *Candida albicans* and, moreover, whether it has clinical relevance, remain challenging to investigate (Fig. 1) [3–5].

Conceptually, clinical investigation of this postulated interaction would manipulate candida colonization among patients and measure the resulting *Pseudomonas* bacteremia incidence [6–8]. Such an investigation would be logistically complex. Additional challenges to such an approach are that the blood stream infection (BSI) endpoints are generally uncommon or rare, the key body site location of any postulated interaction, whether the oropharynx or elsewhere, remains unclear and measuring colonization, whether bacterial or candida, is problematic. Moreover, whether changes in the metabolic activity, hyphal growth or the mere viable count of Candida colonisation drives any interaction towards a propensity for invasive infection remains moot.

A novel approach uses the collective observations from numerous studies of assorted and variously formulated anti-septic, antibiotic, anti-fungal, and non-decontamination-based interventions in the prevention of ICU acquired infections such as ventilator associated pneumonia (VAP) [9–17]. Candidemia and bacteremia incidences, being occasional secondary endpoints within these studies, in association with varying Candida colonisation occurring as a bystander process to the study intervention, serve as a natural experiment.

Selective digestive decontamination (SDD), a widely studied antibiotic-based regimen, combines antibiotic and antifungal exposures. Moreover, the SDD concept invokes population effects which need to be considered in any analysis. Several SDD studies in the ICU setting avoided these anticipated contextual effects by using either non-concurrent or no control group patients [18–22].

The objectives here are threefold. First, to recapitulate the study level evidence for various ICU infection prevention interventions versus each of VAP and BSI with Candida or *Pseudomonas*. Second, to develop models of colonization and infection based on the postulated interaction between Candida and *Pseudomonas* colonization as impacted by various infection prevention interventions by confronting the models using group level infection data using GSEM modelling. Third, to estimate the relative impacts of anti-septic, antibiotic, and specific anti-fungal agents as singular or compound exposures on bacteremia and candidemia within the optimal GSEM model.

## Materials and methods

Being an analysis of published work, ethics committee review of this study was not required.

### Study selection and decant of groups

**Fig. 2:**
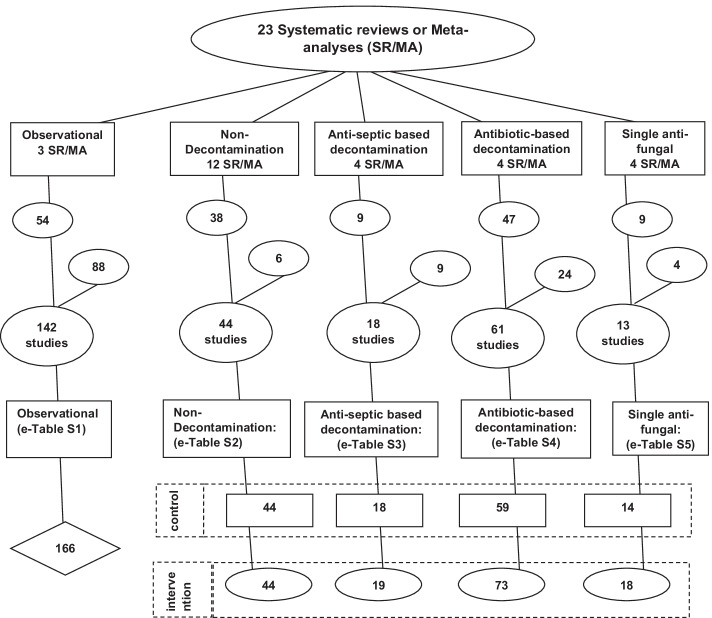
Search method, screening criteria and resulting classification of eligible studies and subsequent decant of component groups. The six steps are as follows: (1) An electronic search for systematic reviews or meta-analysis (SR/MA) containing potentially eligible studies using search terms; “ventilator associated pneumonia”, “mechanical ventilation”, “intensive care unit”, each combined with either “meta-analysis” or “systematic review” up to November 2021; (2) The systematic reviews were then searched for studies of patient populations requiring prolonged (> 24 h) ICU admission (3) The studies were triaged from the systematic reviews into one of five categories; studies in which there was no intervention (observational studies), studies of various non-decontamination methods such as methods delivered either via the gastric route, the airway route or via the oral care route, studies of anti-septic methods, studies of antibiotic-based interventions, and studies of single drug antifungal (SAF) prophylaxis. (4) All studies were reviewed for potentially eligible studies and screened against inclusion and exclusion criteria. Any duplicate or ineligible studies were removed and (5) Studies identified outside of systematic reviews were included; (6) The component groups were decanted from each study being control (rectangles), intervention (ovals) and observation (diamond) groups. The total numbers do not tally as some systematic reviews provided studies in more than one category and some studies provided groups in more than one category and some studies have unequal numbers of control and interventions groups

The literature search and study decant used here is as described previously [23–25] and is detailed in Fig. 2. The key inclusion criterion were as follows: patient groups requiring prolonged (> 24 h) ICU stay within either studies of ICU infection prevention interventions or observational studies without an intervention under study, and group level *Candida,* and *Pseudomonas* infection data was available. The studies were streamed into type of infection prevention intervention, being non-decontamination based, anti-septic based, antibiotic based or single anti-fungal (SAF) based methods. Studies without ICU infection prevention interventions were sourced to provide summary benchmark incidence data. Most of the studies had been cited in systematic reviews with additional studies being found by snowball sampling using the ‘Related articles’ function within Google Scholar [26].

The various study level and group level data were extracted for tabulation and to serve as indicator variables in the GSEM models.

### Visual benchmarking

Scatter plots of VAP and blood stream infections in association with *Candida* and *Pseudomonas* infection were generated to facilitate a visual survey of the entire data versus benchmarks as derived from the observational groups.

### Estimation of summary effect sizes

As study events were rare, summary effect sizes were derived using the Peto’s log odds ratio [27]. The Stata ‘meta’ command was used for deriving summary effect sizes, the associated measures of between-study heterogeneity and the caterpillar plots which display the results of individual studies.

### GSEM models: measurement components

The incidences of VAP and blood stream infections in association with each of *Candida and Pseudomonas* were extracted. As *Candida* is generally not considered a cause of VAP, the count of *Candida* as a respiratory tract (RT *Candida*) isolate among patients with suspected VAP was recorded. These counts were each transformed to proportions using the number of patients with prolonged (> 24 h) ICU stay as the denominator. In the GSEM models, *Candida* and *Pseudomonas* colonization are each latent variables within a measurement model for which the infection count proportions serve as endogenous variables.

### GSEM models: indicator variables

The following data constitute exogenous indicator variables of the GSEM models; origin from trauma ICU’s, being defined here as an ICU with > 50% of admissions being for trauma, whether more than 90% of patients of the group received more than 24 h of MV, and a mean (or median) length of ICU stay (ICU-LOS) for the group greater than 7 days. In the extraction of MV percentages, if this was not stated for any group, the percentage receiving MV was assumed to be less than 90%. In the extraction of ICU-LOS data from the studies, surrogate measures including mean (or median) length of mechanical ventilation were taken if the length of ICU-LOS was not available in order to generate a binary variable of ICU-LOS of greater or less than 7 days.

Also, the presence of any of the following group wide risk factors for candidemia (CRF) and invasive *Candida* infection were noted; liver transplantation or liver failure, use of parenteral nutrition, surgery for intestinal perforation, pancreatitis, and being colonized with *Candida*, however that was defined. Anti-septic exposure included use of agents such as chlorhexidine, povidone-iodine and iseganan regardless of whether the application was to the oropharynx, by tooth-brushing or by body-wash.

Antibiotic-based interventions were classified as follows. Topical antibiotic prophylaxis (TAP) is defined without regard to the specific antibiotic constituents and whether application was to the oropharynx and or gastrointestinal tract. Protocolized parenteral antibiotic prophylaxis (PPAP) is the prophylactic use of parenteral antibiotics as dictated by the study protocol whether to the intervention group alone or to both control and intervention groups (duplex studies). Exposure to anti-fungal prophylaxis was identified whether as a single agent (SAF) or in combination with antibiotic-based interventions as within SDD or selective oropharyngeal decontamination (SOD) regimens.

### Structural equation modelling

In the GSEM models, the VAP and blood stream infection counts as proportion data, serve as the measurement components, the group level exposure parameters serve as the indicator variables and colonization with each of *Candida* and *Pseudomonas*, being represented as latent variables, link the indicator and measurement components.

Three candidate GSEM models were developed. The first two, with and without the inclusion of an interaction terms between the latent variables, being *Candida* colonization and *Pseudomonas* colonization and the third with the addition of concurrent control group membership within an antibiotic-based study as an indicator variable to identify postulated contextual effects.

Because the observations are clustered by study, study identifiers were used in the models to enable generation of robust variance covariance matrices of the coefficient estimate parameters. The GSEM model with the lowest Akaike's information criterion (AIC) score was selected as having parsimony and optimal fit from among the candidate models using the ‘GSEM’ command in Stata (Stata 17, College Station Texas, USA) [28]. The post model predictions were obtained using the Stata command ‘nlcom’ to obtain nonlinear combinations of estimators.

## Results

### Characteristics of the studies

Of the 279 studies identified by the search, most were sourced from 23 systematic reviews (Table 1; Fig. 2; Additional file 1: Tables S1–S5)), most were published between 1990 and 2010 and most had a mean ICU-LOS exceeding seven days. Twelve studies had more than one type of intervention group and 15 studies had either more than one or no control group. The majority of groups from studies of infection prevention interventions had less than 150 patients per group versus more than 150 patients in the observational studies.

**Table 1:** Characteristics of studies

	Observational	Non-decontamination	Topical anti-septic^a^	Antibiotic based^b^	Single anti-fungal^c^
*Study characteristics*					
Listing	Additional file 1: Table S1	Additional file 1: Table S2	Additional file 1: Table S3	Additional file 1: Table S4	Additional file 1: Table S5
Number of studies^d^	142	44	18	61	13
MV for > 48 h for < 90%^e^	41	0	9	16	6
PPAP for control groups	0	0	0	10	0
Trauma ICUs^f^	25	8	3	13	1
CRF as selection criteria^g^	11	0	0	11	6
Paediatric ICU			1	1	
North American ICU	36	10	8	6	3
Study publication year (range)	1987–2019	1987–2017	2000–2018	1984–2021	1994–2014
*Group characteristics*					
Number of groups^d^	166	88	37	131	32
Numbers of patients per study group; median (IQR)^h^	280 118–596	75 61–143	130 72–347	47 31–72	69 49–78
Mean length of stay < 7 days; (number of groups)	27	14	12	14	2
Candidemia risk factors; (number of groups)	11	0	0	21	14
*Indicative intervention effect size (VAP / RT candida)*^*i j*^					
VAP Pseudomonas prevention effect (Additional file 1: Fig. S3) (odds ratio; 95% CI; n)	NA	0.75; 0.61–0.91 (39)	0.61; 0.38–0.97 (11)	0.33; 0.26–0.42 (39)	NR
RT candida prevention effect (Additional file 1: Fig. S5) (odds ratio; 95% CI; n)	NA	0.62; 0.42–0.9 (19)	0.37; 0.11–1.29 (8)	0.54; 0.27–1.08 (15)	NR
*Indicative intervention effect size*^*i,k*^* (Bacteremia/Candidemia)*					
Pseudomonas bacteremia prevention effect (Additional file 1: Fig. S4) (odds ratio; 95% CI; n)	NA	7.46; 0.47–120 (1)	1.0 0.67–1.5 (7)	0.82; 0.52–1.29 (19)	NR
Candidemia prevention effect (Additional file 1: Fig. S6) (odds ratio; 95% CI; n)	NA	1.01; 0.06–16.1 (1)	0.75 0.55–1.03 (7)	0.48; 0.27–0.85 (17)	0.43; 0.23–0.8 (16)^l^

^a^Among anti-septic studies, topical chlorhexidine was used in 15 of 20 intervention groups

^b^Among TAP intervention groups, the most common antibiotic combination used were polymyxin in combination with an aminoglycoside in 62 of 84 groups. Also, a topical anti-fungal was used in all but eight interventions groups, with amphotericin being the most common anti-fungal (50 intervention groups)

^c^Fluconazole was the most common single agent antifungal, used in seven intervention groups

^d^Note, several studies had more than one control and or intervention group. Hence the number of groups does not equal the number of studies

^e^Studies for which less than 90% of patients were reported to receive > 48 h of MV

^f^Trauma ICU arbitrarily defined as an ICU with more than 50% of admissions for trauma

^g^Use of Candidemia risk factors (CRF) as study inclusion criteria

^h^Data is median and inter-quartile range (IQR)

^i^Note that studies with zero events in both control and intervention arms do not contribute in the calculation of summary effect size

^j^Effect size is indicative for each category. Anti-septic interventions include Iseganin in one study; TAP interventions were usually in combinations with an anti-fungal agent; SAF interventions were single include nystatin and TAP in one study and fluconazole in combinations with TAP in another study

^k^Effect size is indicative as several interventions with combinations of agents have been included. TAP interventions were usually in combinations with an anti-fungal agent (most commonly amphotericin); SAF interventions were either nystatin (six intervention groups) or fluconazole or another agent (nine intervention groups)

^l^Summary effect size from 7 studies that used nystatin was 1.2 (0.79–1.83) and from 9 studies that used an azole as SAF was 0.21 (0.11–0.4)

**Fig. 3:**
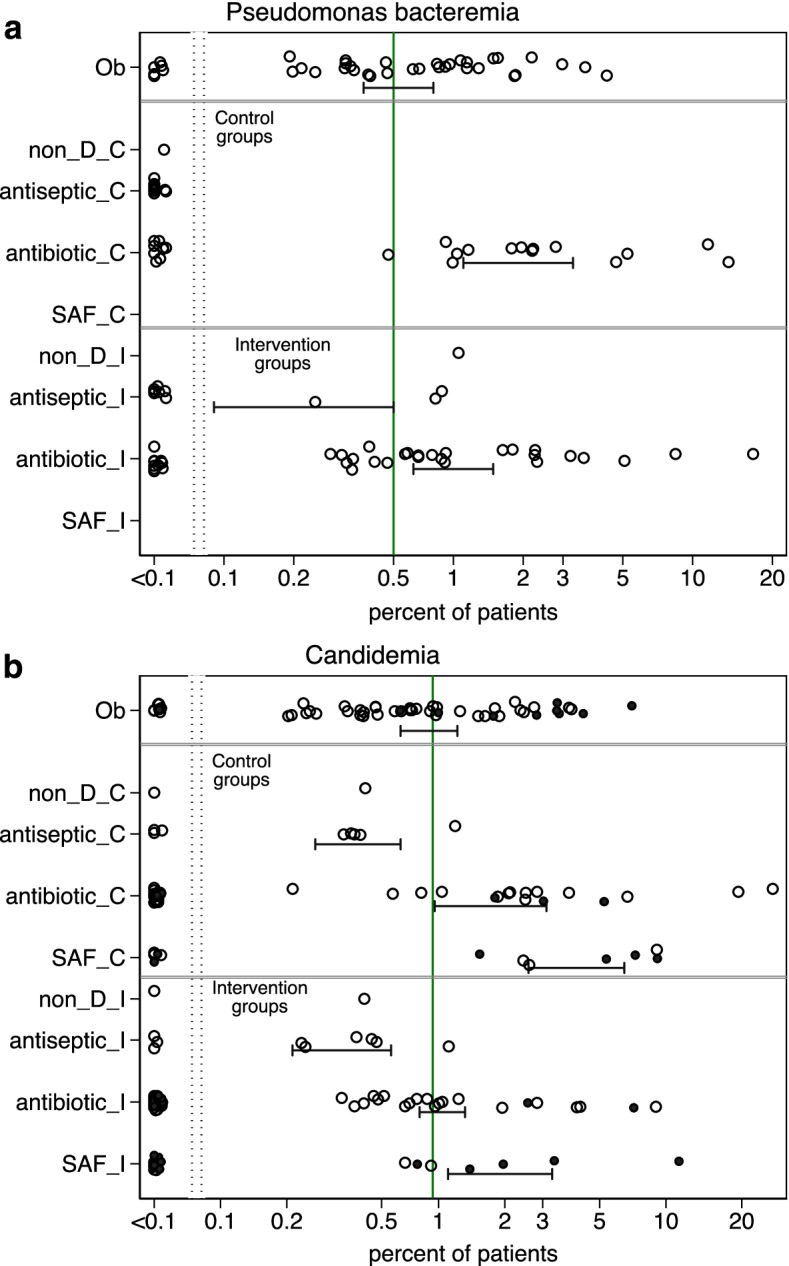
**a**, **b** Scatter plots, on a logit scale, of the incidence proportions of *Pseudomonas* bacteremia (**a**) and candidemia (**b**) for groups from 289 studies as listed in Additional file 1: Tables S1 to S5. The mean proportion (and 95% CI) derived by random effect meta-analysis for each category of component (observational [Ob], control [_C] and intervention [_I]) group derived from observational [Ob], non-decontamination (non-D), antibiotic-based and single anti-fungal (SAF) studies, is displayed. In each plot, the benchmark proportion (solid vertical line) is the mean proportion derived from the observational groups. Those component groups that did (solid symbols) versus did not (open symbols) select patients with CRF’s are indicated. *NCC* non-concurrent control, *CC* concurrent control. Note that antibiotic groups received multiple exposures in association with compound regimens (e.g. SDD and SOD, which combine TAP, an antifungal together with or without PPAP)

The incidence of BSI (Fig. 3) and VAP (Additional file 1: Figs. S1, S2) with each of *Candida* and *Pseudomonas* ranged approximately 100-fold across the various observation, control and intervention groups of the 279 studies. In each case, the incidence was approximately 50% or more higher among concurrent control groups within studies where intervention groups received TAP versus a benchmark derived from observational groups. The candidemia incidence was generally higher among groups from SAF studies as patient selection for most of these studies was commonly based on presence of CRF.

Bacteremia and candidemia incidences were generally lower among studies of anti-septic interventions as there was a lower proportion with respect to each of the following versus other study categories: mean length of stay more than 7 days, trauma ICU’s, patient selection for CRF and use of MV for > 48 h.

### Summary effect sizes

Among the summary prevention effects, the strongest was for antibiotic-based interventions versus *Pseudomonas* VAP (odds ratio 0.33; 95% CI; 0.26 to 0.42) (Table 1, Additional file 1: Fig S3). In the prevention of candidemia, the indicative summary effect size for the SAF and antibiotic-based interventions were similar. All other summary effect estimates for all other interventions versus the VAP, bacteremia, or candidemia end-points were either weaker or not significant (Table 1; Additional file 1: Table S6; Fig S3–S6). Of note, surprisingly, no significant prevention effect for *Pseudomonas* bacteremia was apparent for any intervention (Table 1; Additional file 1: Fig S4).

### GSEM modelling

**Fig. 4:**
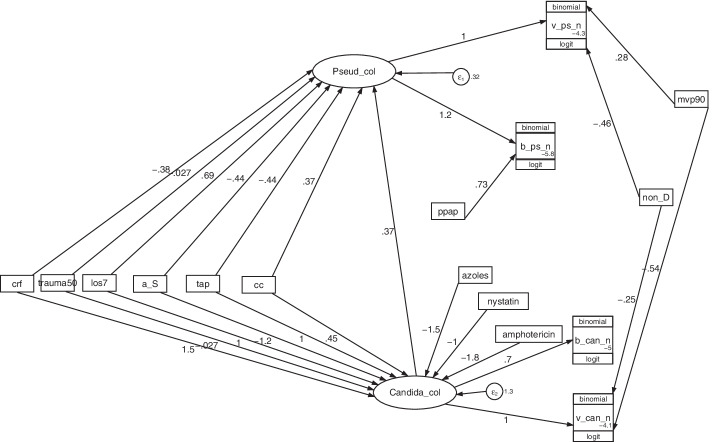
GSEM of the interaction model in relation to *Pseudomonas* and *Candida* infection data. *Candida* col and *Pseudomonas* col (ovals) are latent variables representing *Candida* and *Pseudomonas* colonization, respectively. The variables in rectangles are binary predictor variables representing the group level exposure to the following; patient selection for candidemia risk factors (CRF); trauma ICU setting (trauma50), mean or median length of ICU stay ≥ 7 days (los7), exposure to a topical anti-septic (a_S), exposure to TAP (tap), concurrency of a control group with an antibiotic-based intervention group (CC), exposure to a non-decontamination based prevention method (non-D), use of mechanical ventilation for more than 90% (mvp90) or exposure to PPAP (ppap). Note that the model factorizes exposures from compound regimens (e.g. SDD and SOD, which combine TAP, an antifungal together with or without PPAP) into singleton TAP, PPAP and anti-fungal exposures. The circles contain error terms. The three part boxes represent the binomial data for *Candida* and *Pseudomonas* VAP (v_can_n, v_ps_n) and candidemia (b_can_n) or bacteremia (b_ps_n) counts with the number of patients as the denominator which is logit transformed using the logit link function in the generalized model

Three GSEM models of the relationship between various group level exposures on *Candida* and *Pseudomonas* colonization as latent variables were evaluated for fit and parsimony (Table 2; Additional file 1: Fig S7–S8; Fig. 4). The introduction of firstly *Candida* colonization as a cofactor towards *Pseudomonas* colonization (Model C to Model B), and then, addition to the model of membership of a concurrent control group within a study of an antibiotic-based interventions as an indicator variable (Model B to Model A), each improved the model fit towards the optimal model (Fig. 4).

**Table 2:** Development of GSEM models; model C, model B and model A

	Model C	Model B	Model A	
	Additional file 1: Fig S7	Additional file 1: Fig S8	Fig. 4	
				95%CI
*Factor*^*a−i*^				
**b_Ps_n**				
Pseudomonas colonization	1.25***	1.23***	1.23***	0.86 to 1.59
ppap	0.76*	0.74*	0.73*	0.1 to 1.36
_cons	− 5.92***	− 5.79***	− 5.83***	− 6.37 to − 5.28
**v_Ps_n**				
Pseudomonas colonization	1	1	1	(constrained)
mvp90	0.35	0.25	0.28	− 0.11 to 0.67
non_D	− 0.54***	− 0.52***	− 0.46***	− 0.71 to − 0.22
_cons	− 4.48***	− 4.22***	− 4.31***	− 5.01 to − 3.57
*Pseudomonas colonization*				
cc			0.37*	0.06 to 0.68
tap	− 0.64***	− 0.49***	− 0.44***	− 0.68 to − 0.21
Anti-septic (a_S)	− 0.86***	− 0.44	− 0.44	− 0.94 to 0.06
los7	0.82***	0.69***	0.69***	0.38 to 0.99
Trauma (trauma50)	− 0.05	− 0.01	− 0.03	− 0.4 to 0.34
crf	0.20	− 0.29	− 0.38	− 0.95 to 0.19
Candida colonization		0.39***	0.37***	0.26 to 0.49
**b_can_n**				
Candida colonization	0.73***	0.7***	0.7***	0.33 to 1.07
_cons	− 5.01***	− 4.95***	− 4.97***	− 5.34 to − 4.6
**v_can_n**				
Candida colonization	1	1	1	(constrained)
mvp90	− 0.77	− 0.56	− 0.54	− 1.4 to 0.31
non_D	− 0.22	− 0.31	− 0.25	− 0.84 to 0.33
_cons	− 3.65***	− 4.0***	− 4.05***	− 5.77 to − 2.36
*Candida colonization*				
cc			0.45	− 0.19 to 1.09
tap	0.96*	0.96*	1.02*	0.11 to 1.93
Anti-septic (a_S)	− 1.28**	− 1.27**	− 1.23**	− 2.13 to − 0.32
los7	0.13	0.13	0.1	− 0.45 to 0.65
Trauma (trauma50)	0.11	0.02	0.03	− 0.87 to 0.82
crf	1.43**	1.51**	1.48**	0.45 to 2.5
Amphotericin	− 1.73**	− 1.77***	− 1.78***	− 2.79 to − 0.78
Nystatin	− 0.90	− 1.05	− 1.04	− 2.33 to 0.3
Azoles and other	− 1.44**	− 1.53**	− 1.47**	− 2.56 to − 0.38
**Error terms**				
var (e. Pseudomonas col)	0.52***	0.33***	0.32***	0.23 to 0.44
var (e. Candida col)	1.37***	1.3***	1.27***	0.82 to 1.97
**Model fit**^i^				
AIC	3974	3928	3921	–
Groups (n)	464	464	464	–
Clusters (n)	279	279	279	
Factors (N)	29	30	32	–

Shown in this table are models derived with all studies increasing in complexity from left to right. The figures corresponding to models C (Additional file 1: Fig. S7), model B (Additional file 1: Fig. S8) models A (Fig. 4)

*p < 0.05; **p < 0.01; ***p < 0.001

^a^v_ps_n is the count of *Pseudomonas* VAP; v_can_n is the count of *RT Candida*; b_ps_n is the count of *Pseudomonas* bacteremia and b_can_n is the count of Candidemia

^b^ppap is the group wide use of protocolized parenteral antibiotic prophylaxis; tap is topical antibiotic prophylaxis; non-D is a non-decontamination intervention

^c^mvp90 is use of mechanical ventilation by more than 90% of the group

^d^crf is group wide exposure to a candidemia risk factor

^e^LOS7 is a mean or median length of ICU stay for the group of more than 7 days

^f^Trauma ICU arbitrarily defined as an ICU for which > 50% of admissions were for trauma

^g^*Pseudomonas* colonization (*Pseudomonas* col) is a latent variable

^h^*Candida* colonization (*Candida* col) is a latent variable

^i^Model fit; AIC is Akaike’s information criteria. This indicates model fit taking into account the statistical goodness of fit and the number of parameters in the model. Lower values of AIC indicate a better model fit. Groups (n) is the number of patient groups; clusters (n) is the number of studies; factors (N) is the number of parameters in the model

In the optimal model (Model A), the coefficients for singular exposure to anti-septic agents (− 1.23; − 2.1 to − 0.32), amphotericin (− 1.78; − 2.79 to − 0.78) and topical antibiotic prophylaxis (TAP; + 1.02; + 0.11 to + 1.93) versus *Candida* colonization were similar in magnitude but contrary in direction.

In all models, group wide exposure to CRF, anti-septics and singular exposures to each of TAP and the antifungals, although less so for nystatin, displayed strong and significant associations with the Candida colonization latent variable, and these were generally consistent across the three models. By contrast, the same exposures versus *Pseudomonas* colonization were generally weaker, less consistent between models and variably significant.

Of note, the size of the association between *Pseudomonas* colonization and, on the one hand, membership of a concurrent control group within a study of antibiotic-based interventions, and on the other, with exposure to TAP, were similar in magnitude but contrary in direction and significance. By contrast, the size of these two factors on Candida colonization were similar in direction but differed in magnitude and significance.

### Post GSEM modelling predictions

**Fig. 5:**
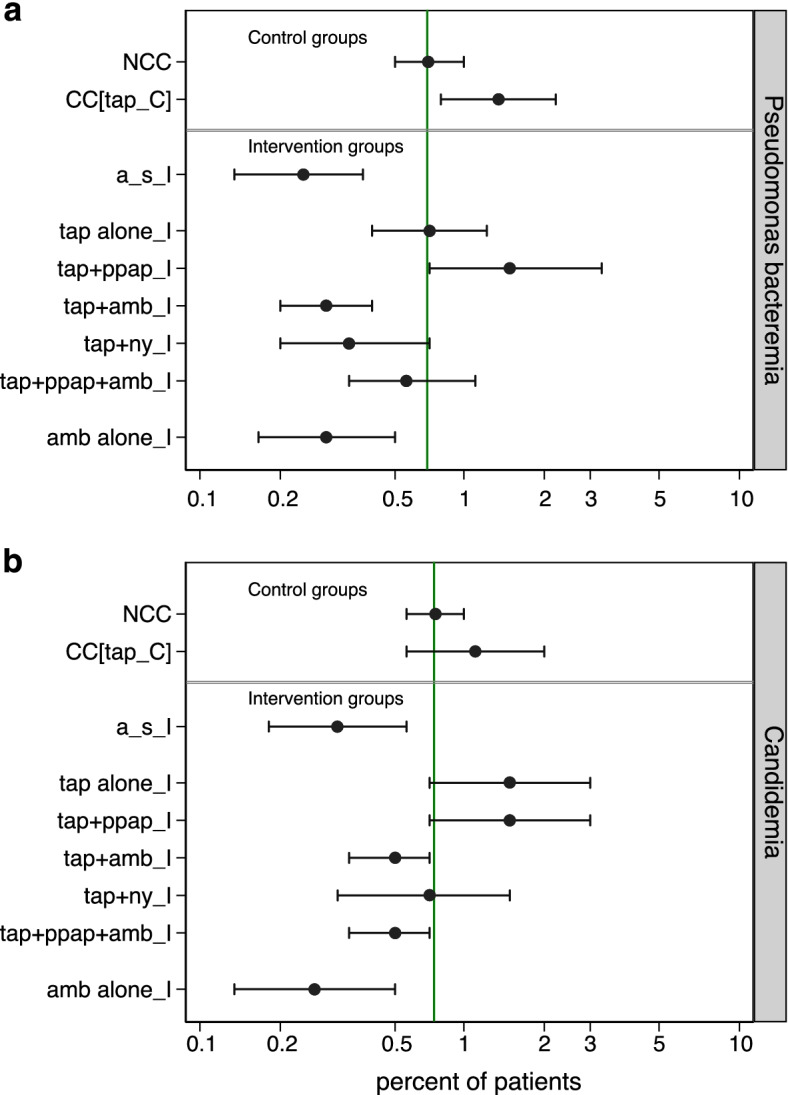
**a**, **b** Model predictions derived from model A (Fig. 4) for the incidence proportions of *Pseudomonas* bacteremia (**a**), and candidemia (**b**) for a putative group of patients in a non-trauma ICU with mean LOS > 7 days without selection for CRF. The projections are for control (top) or intervention (bottom panel) groups receiving prophylaxis with various singleton or combination interventions. In each plot, the benchmark proportion (solid vertical line) is the mean prediction derived for an equivalent NCC group without exposures. *NCC* non-concurrent control, *CC* concurrent control, *non-D* non-decontamination, *a_s* anti-septic, *TAP* topical antibiotic prophylaxis, *amb* amphotericin, *ny* nystatin, *ppap* protocolized parenteral antibiotic prophylaxis

Post model predictions of *Pseudomonas* bacteremia and Candidemia incidences were estimated for a putative group of non-trauma ICU patients with group mean LOS greater than seven days and without patient selection for CRF. Predictions were made for various combination and singleton group wide exposures to anti-septic agents, TAP, PPAP, nystatin and amphotericin versus a benchmark derived for an equivalent putative non-concurrent control group (Fig. 5). In every case, singleton exposure to either amphotericin or to anti-septics outperformed singleton exposure to TAP towards lower predicted bacteremia incidences. Exposure to TAP combined with amphotericin, but not nystatin, was associated with significantly lower predicted bacteremia incidences versus benchmark.

## Discussion

There were three objectives here. First, the study level evidence for various ICU infection prevention interventions toward preventing VAP and BSI derived here broadly recapitulates prior summary estimates among systematic reviews in the literature (Additional file 1: Table S6) [9–17]. The effect sizes are indicative as each category includes broadly selected studies, with some having compound interventions, such as TAP combined with an anti-fungal, together with or without PPAP given to either or both of control and intervention groups in antibiotic-based studies. The selection of studies in the summaries here is congruous with that in the literature summaries. Of note, the concurrent control groups within antibiotic-based intervention studies have unusually high incidences for several end-points that are unexplained in previous meta-regression models, taking into account several other group level variables not included here, such as year of publication, and likely represents a strong contextual effect [29, 30].

The second objective is the development of GSEM models. The optimal model includes both the postulated interaction between Candida and *Pseudomonas* colonization and the contextual influences of TAP on concurrent control groups. In confronting the optimal GSEM model with published group level infection data, the anti-fungal agents, such as azoles and amphotericin, and the anti-septic agents, each showed strong prevention effects versus Candida colonization. By contrast, TAP as a singular exposure versus *Pseudomonas* colonization and versus Candida colonization demonstrated effects that were weaker and variable in direction and significance.

Thirdly, in post GSEM model predictions, the estimated effects of singular exposure to topical amphotericin and anti-septic agents would each more than halve the incidence of candidemia and *Pseudomonas* bacteremia, although for absolute differences being approximately one percentage point or less. These differences would be challenging to detect. For example, a cluster-randomized trial demonstrating halving in *Pseudomonas* bacteremia incidence from 1% in the control group to 0.5% in the intervention group would need to enrol over 2000 ICU’s each providing 500 patients per arm to provide 80% power.

By contrast, singular TAP exposure, with or without PPAP in combination, was either neutral or promoted *Pseudomonas* bacteremia and candidemia incidence. Of note, the most common PPAP among SDD studies is cefotaxime, which is not generally active against *Pseudomonas*. That antibiotic exposure in the ICU environment paradoxically promotes *Pseudomonas* acquisition is a finding with precedent [31, 32]. Moreover, bacterial colonization rebounds following cessation of TAP with effects on the whole-of-ICU population [33]. Whether rebound contributes to colonization pressure, and the contextual effects of TAP exposure, needs to be further examined [34].

The observations here are paradoxical. On the one hand, antibiotic-based interventions (being in combination with anti-fungal agents as used within studies of SDD) appear to show strong prevention effects against VAP that is most apparent for *Pseudomonas* VAP together with the appearance of a halving in candidemia among studies of antibiotic-based interventions.

On the other hand, despite these two strong prevention effects of antibiotic-based interventions, there is insignificant prevention of *Pseudomonas* bacteremia. Moreover, the incidences of candidemia and *Pseudomonas* bacteremia are generally higher among the concurrent control groups of antibiotic-based studies and reflect the contextual effects.

The interaction underlying these paradoxical observations, potentially explains how ICU-acquired gram-negative bacteremia might occur seemingly without preceding colonization [18, 35, 36]. They also could account for the bacteremia prevention effects observed in large studies of antibiotic-based interventions of SDD regimens containing topical polymyxin and tobramycin combined with amphotericin as the anti-fungal [20, 21], whereas the largest SDD study, where the same TAP regimen was combined with nystatin [22], failed to demonstrate any prevention effects against any bacteremia, or candidemia, end-points.

The SEM technique is an emerging method among critical care and infection pathogenesis research that enables group level modelling of multiple simultaneously observed variables [37, 38]. This technique enables the testing of causal concepts that might underlie relationships between observed variables mediated through latent variables. GSEM allows generalized linear response functions in addition to the linear response functions allowed by non-generalized SEM. A strength of GSEM modelling is the ability to incorporate observations from clusters with missing observations under the assumption of missing at random. This enables the inclusion of groups from studies either lacking control groups or providing data for only some end-points.

### Limitations

There are multiple limitations and cautions in the interpretation of the modelling here. The GSEM is a group level modelling of two latent variables, Candida and *Pseudomonas* within a postulated model of interaction. These latent variables and the coefficients derived in the GSEM are indicative only. They have no counterpart at the level of any one patient or study and cannot be directly measured.

The second limitation is that there was no ability nor purpose to adjust for the underlying patient level risk. There was considerable heterogeneity in the interventions, populations, and study designs among the studies, which were conducted up to three and a half decades ago, meeting intentional broad inclusion criteria. This stands in contrast to the technique of network meta-analysis (NMA) applied to data obtained from studies selected according to tight inclusion criteria towards satisfying transitivity assumptions. With transitivity, a NMA enables comparisons of multiple interventions as allocated to comparable patient groups within randomized controlled trials towards estimating patient level effects on a defined end point from study level data. By contrast, the GSEM technique enables causal modelling of group-level associations towards deriving group level inferences among patient groups experiencing compound exposures within studies assembled with less stringent inclusion criteria. This allows the inclusion of concurrent control groups from antibiotic-based studies where contextual effects invalidate the transitivity assumption.

The third limitation is that the GSEM model is deliberately simplistic. There are only limited numbers of key group level factors, the exposures are entered as binary variables, and with interaction between the latent variables being the only interactions tested. In reality, the relationships between expoures and outcomes will likely be graded and complex with potentially compound expoure interactions.

The impact of anti-fungal prophylaxis on *Pseudomonas* colonization and infection is the only bacterial species examined here. However, other bacterial species, such as Acinetobacter warrant examination given the observed paradoxical incidences that occur in association within studies of topical antibiotic prophylaxis [39, 40].

Finally, the various regimens of antibiotic-based, anti-septic and anti-fungal interventions used within the various studies have been considered as similar within each category. This is a deliberate simplification. For example, some SAF interventions were administered parenterally rather than topically. In addition, the intensity and duration of application, and the body site targeted by the various interventions, varied among the studies and have not been modelled. On the other hand, a strength of this analysis is that the various compound interventions, as for example within SDD regimens comprising TAP, PPAP, and anti-fungal components, are factorized towards estimating their separate singleton associations on the latent variables within the GSEM model.

## Conclusion

GSEM modelling of *Pseudomonas* and candida colonization, each as latent variables versus group level exposures, demonstrates complex and paradoxical relationships that would not be apparent in any single study examined in isolation nor within a summary effect of the collective studies as derived by conventional meta-analytic modelling. The model provides support to the postulated interaction between candida and *Pseudomonas* colonization in facilitating invasive *Pseudomonas* infections. Anti-fungal interventions have potentially stronger prevention impact on *Pseudomonas* bacteremia, mediated via candida colonization, than does singleton TAP or PPAP exposures, which either have no impact or which promote *Pseudomonas* bacteremia.

## Supplementary Information

**Additional file 1: Table S1.** Observational studies (Benchmark groups). **Table S2.** Groups of non-decontamination studies. **Table S3.** Groups of anti-septic studies. **Table S4.** Groups of antibiotic-based (= TAP ± PPAP ± antifungal) studies. **Table S5.** Groups from single drug anti-fungal (SAF) studies. **Table S6.** Review of effect sizes in the literature. **Fig S1.** a & b. *Pseudomonas*; VAP and bacteremia count data. **Fig S2**. a & b. Candidemia and RT candida count data. **Fig S3.** Effect size VAP *Pseudomonas. ***Fig S4.** Effect size *Pseudomonas* bacteremia. **Fig S5.** Effect size RT* candida. ***Fig S6.** Effect size Candidemia. **Figs. S7, S8.** GSEM model C & GSEM model B.

## Data Availability

All data generated or analysed during this study are included in this published article and the online Additional material (ESM).

## Abbreviations

BSI: Blood stream infection
CRF: Candida risk factors
ICU: Intensive care unit
MV: Mechanical ventilation
SAF: Single anti-fungal
VAP: Ventilator associated pneumonia
PPAP: Protocolized parenteral antibiotic prophylaxis
RT Candida: Respiratory tract Candida
SDD: Selective digestive decontamination
TAP: Topical antibiotic prophylaxis
GSEM: Generalized structural equation models
