# Supplementary material for: Structural equation modelling the relationship between anti-fungal prophylaxis and Pseudomonas bacteremia in ICU patients
Source: Intensive Care Med Exp. 2022 Jan 21;10:2. doi: 10.1186/s40635-022-00429-8 (PMC8776977; doi:10.1186/s40635-022-00429-8)
Supplement: Supplementary file 1 — Additional file 1: Table S1. Observational studies (Benchmark groups). Table S2. Groups of non-decontamination studies. Table S3. Groups of anti-septic studies. Table S4. Groups of antibiotic-based (= TAP ± PPAP ± antifungal) studies. Table S5. Groups from single drug anti-fungal (SAF) studies. Table S6. Review of effect sizes in the literature. Fig S1. a & b. Pseudomonas; VAP and bacteremia count data. Fig S2. a & b. Candidemia and RT candida count data. Fig S3. Effect size VAP Pseudomonas. Fig S4. Effect size Pseudomonas bacteremia. Fig S5. Effect size RT candida. Fig S6. Effect size Candidemia. Figs. S7, S8. GSEM model C & GSEM model B. [file 40635_2022_429_MOESM1_ESM.docx]

*Candida*-Pseudomonas-interaction modelled within ICU infection prevention studies

James C Hurley, MB BS, D Med Sci, M Epi, PhD, FRACP;

Author affiliations;

• Associate Professor, Melbourne Medical School, University of Melbourne,

• and Physician, Division of Internal Medicine, Ballarat Health Services, Ballarat, Victoria, Australia. e-mail [jamesh@bhs.org.au](mailto:drjch@ncable.net.au)

Supplemental file contents:

Table S1: Observational studies (Benchmark groups) 2 - 6

Table S2: Groups of non-decontamination studies 7- 10

Table S3: Groups of anti-septic studies 11 – 12

Table S4: Groups of antibiotic-based (= TAP ± PPAP ± antifungal) studies 13 – 17

Table S5: Groups from single drug anti-fungal (SAF) studies 18

Table S6: Review of effect sizes in the literature 19

References 20 – 38

Fig S1 a & b. *Pseudomonas*; VAP and bacteremia count data 39

Fig S2 a & b. Candidemia and RT candida count data 40

Fig S3 Effect size VAP *Pseudomonas*  41

Fig S4 Effect size *Pseudomonas* bacteremia 42

Fig S5 Effect size RT *candida*  43

Fig S6 Effect size Candidemia 44

Fig S7 & S8 GSEM model C & GSEM model B 45

Abbreviations;

BSI, blood stream infection; CRF, Candida risk factors; ICU, Intensive Care Unit; MV, Mechanical Ventilation ; SAF, single anti-fungal; VAP ventilator associated pneumonia; PPAP, Protocolized parenteral antibiotic prophylaxis; RT Candida; Respiratory tract Candida; SDD, Selective Digestive Decontamination; TAP, topical antibiotic prophylaxis; GSEM, generalized structural equation models.

**Table S1: Observational studies (Benchmark groups) ^a^**

| Author | Ref | Notes | MV | LOS | Patients | VAP | | |  | Blood stream infection | | |
| --- | --- | --- | --- | --- | --- | --- | --- | --- | --- | --- | --- | --- |
|  |  |  | % | d | n | v_ps_n |  | v_can_n |  | b_ps_n |  | b_can_n |
| A'court 1993 | 1 | T | 100 | 12 | 150 | 17 |  |  |  | 6 |  |  |
| Alvarez-Lerma 1996 | 2 |  | 93 | 7 | 6494 | 174 |  |  |  |  |  |  |
| Antonelli 1994 | 3 | T | 70 | 17 | 124 | 5 |  | 1 |  |  |  |  |
| Apostolopoulou 2003 | 4 |  | 100 | 16 | 175 | 17 |  |  |  |  |  |  |
| Arumugam 2018 | 5 | T | 100 | 7 | 332 | 7 |  |  |  |  |  |  |
| Azoulay noCandida 2006 | 6 |  | 100 | 17 | 589 | 30 |  |  |  |  |  | 4 |
| AzoulayCandida 2006 | 6 | crf | 100 | 17 | 214 | 19 |  |  |  |  |  | 2 |
| Bailly 2015 | 7 |  | 100 | 30 | 1491 |  |  |  |  |  |  | 11 |
| Bekaert 2011 | 8 |  | 100 | 8 | 4479 | 155 |  |  |  |  |  |  |
| Bercault 2001 | 9 |  | 100 | 26 | 1144 |  |  | 2 |  |  |  |  |
| Bercault_IHT 2005 | 10 |  | 100 | 9 | 118 | 9 |  |  |  |  |  |  |
| Bercault_noINT 2005 | 10 | I | 100 | 11 | 118 | 3 |  |  |  |  |  |  |
| Berrouane_all 1998 | 11 | T | 83 | 13 | 565 | 40 |  |  |  |  |  |  |
| Blot >74 2014 | 12 |  | 100 | 8 | 516 | 10 |  |  |  |  |  |  |
| Blot 45_64 2014 | 12 |  | 100 | 8 | 670 | 20 |  |  |  |  |  |  |
| Blot 65_74 2014 | 12 |  | 100 | 8 | 549 | 20 |  |  |  |  |  |  |
| Bochicchio 2004 | 13 | T | 100 | 13 | 678 | 22 |  |  |  |  |  |  |
| Bonten'94 1994 | 14 |  | 100 | 25 | 64 | 6 |  |  |  |  |  |  |
| Boots 2008 | 15 |  | 100 | 13 | 412 | 15 |  | 0 |  |  |  |  |
| Bornstain 2004 | 16 |  | 100 | 12 | 747 | 23 |  |  |  |  |  |  |
| Borzotta 1999 | 17 | T | 85 | 10 | 459 |  |  |  |  |  |  | 7 |
| Braun 1986 | 18 | T | 100 |  | 66 | 0 |  |  |  |  |  |  |
| Bregeon 1997 | 19 |  | 100 |  | 660 | 33 |  | 3 |  |  |  |  |
| Bronchard 2004 | 20 | T | 100 | 23 | 109 | 0 |  |  |  |  |  |  |
| Cade 1993 | 21 |  | 98 | 16 | 98 | 4 |  | 5 |  | 1 |  | 1 |
| Cavalcanti 2006 | 22 | T | 100 | 10 | 190 | 9 |  | 6 |  |  |  |  |
| Cenderero 1999 | 23 |  | 100 | 7 | 123 | 4 |  | 0 |  |  |  |  |
| Chaari 2015 | 24 | T | 100 | 8 | 175 | 20 |  |  |  |  |  |  |
| Charles 2005 | 25 | crf | 97 | 21 | 56 |  |  |  |  |  |  | 1 |
| Charles 2005 | 25 |  | 75 | 14 | 36 |  |  |  |  |  |  | 0 |
| Chastre 1998 | 26 |  | 100 | 14 | 243 | 36 |  | 7 |  |  |  |  |
| Chevret 1993 | 27 |  | 100 | 5 | 255 | 21 |  | 4 |  |  |  |  |
| Combes 2000 | 28 | T | 100 | 18 | 104 | 0 |  |  |  |  |  |  |
| Cook_non-trauma 2010 | 29 |  | 100 | 13 | 2080 | 7 |  | 4 |  |  |  |  |
| Cook_trauma 2010 | 29 | T | 100 | 8 | 511 | 16 |  | 1 |  |  |  |  |
| Craven-medical 1988 | 30 |  | 100 | 6 | 277 | 9 |  | 1 |  | 1 |  |  |
| Craven-surgical 1988 | 30 |  | 100 | 6 | 521 | 17 |  | 5 |  | 6 |  |  |
| Daschner 1988 | 31 |  | 100 | 6 | 116 | 9 |  | 4 |  |  |  |  |
| De waele 2003 | 32 | crf | 79 | 16 | 46 |  |  |  |  |  |  | 0 |
| de_Latorre 1995 | 33 |  | 100 | 15 | 80 | 7 |  | 2 |  |  |  |  |
| de_Santis 2000 | 34 |  |  |  | 713 |  |  |  |  | 3 |  |  |

**Table S1 (continued): Observational studies (Benchmark groups)**

| Author | Ref | Notes | MV | LOS | Patients | VAP | | |  | Blood stream infection | | |
| --- | --- | --- | --- | --- | --- | --- | --- | --- | --- | --- | --- | --- |
|  |  |  | % | d | n | v_ps_n |  | v_can_n |  | b_ps_n |  | b_can_n |
| de_Santis 2013 | 34 |  |  |  | 1318 |  |  |  |  | 1 |  |  |
| El-Masri 2004 | 35 | T |  | 11 | 361 |  |  |  |  | 1 |  |  |
| Ensminger 2006 | 36 | C | 100 | 7 | 92 | 2 |  |  |  |  |  |  |
| Ertugrul 2006 | 37 | T | 100 | 10 | 100 | 3 |  |  |  | 0 |  |  |
| Esteve 2007 | 38 |  | 80 | 16 | 395 |  |  |  |  | 8 |  |  |
| Esteve 2007 | 38 |  | 78 | 17 | 404 |  |  |  |  | 4 |  |  |
| Evans 2010 | 39 | T | 100 | 8 | 416 | 18 |  |  |  |  |  |  |
| Ewig 1999 | 40 | T | 100 | 10 | 48 | 4 |  | 1 |  |  |  |  |
| Fabian_all 1993 | 41 | T | 100 | 11 | 278 | 10 |  |  |  |  |  |  |
| Fagon'89 1989 | 42 |  | 100 | 13 | 567 | 16 |  |  |  |  |  |  |
| Ferreira 2015 | 43 |  | 94 | 5 | 2527 |  |  |  |  |  |  |  |
| Gacouin 2009 | 44 |  | 100 | 11 | 361 | 21 |  |  |  |  |  |  |
| Garcı´a-Garmendia 2001 | 45 |  | 46 | 5 | 2640 |  |  |  |  | 14 |  | 23 |
| Garrouste-Orgeas 1997 | 46 |  | 100 | 11 | 86 | 9 |  | 1 |  |  |  |  |
| Garrouste-Orgeas 2006 | 47 |  | 75 | 11 | 3247 |  |  |  |  | 19 |  |  |
| George 1998 | 48 |  | 100 | 8 | 223 | 6 |  | 2 |  |  |  |  |
| Georges 2000 | 49 |  | 100 | 20 | 135 | 19 |  |  |  |  |  |  |
| Giamarellos-Bourboulis  2009 | 50 | T | 100 | 12 | 72 | 5 |  | 2 |  | 1 |  |  |
| Giard 2008 | 51 |  | 100 | 9 | 7236 | 168 |  |  |  |  |  |  |
| Gruson-97-98 2000 | 52 | I | 100 |  | 1029 | 47 |  |  |  |  |  |  |
| Gruson-95-96 2000 | 52 |  | 100 |  | 1004 | 62 |  |  |  |  |  |  |
| Gruson-99-01 2003 | 53 |  | 100 |  | 823 | 41 |  |  |  |  |  |  |
| Guérin 1997 | 54 |  | 100 | 19 | 260 | 14 |  | 0 |  |  |  |  |
| Gursel 2010 | 55 |  | 100 | 10 | 92 | 10 |  | 1 |  |  |  |  |
| Heyland 1999 | 56 |  | 100 | 7 | 1014 | 38 |  | 26 |  |  |  |  |
| Holzapfel_93 1993 | 57 |  | 100 | 10 | 300 | 0 |  |  |  | 0 |  |  |
| Huang_1pre 2013 | 58 |  |  | 3 | 15816 |  |  |  |  | 5 |  | 38 |
| Huang_1SC 2013 | 58 |  |  | 3 | 23480 |  |  |  |  | 14 |  | 49 |
| Hugonnet 2007 | 59 |  | 100 | 6 | 936 | 31 |  | 40 |  |  |  |  |
| Hyllienmark 2007 | 60 |  | 100 | 4 | 221 | 0 |  |  |  |  |  |  |
| Hyllienmark 2013 | 61 | T | 42 | 3 | 135 | 4 |  |  |  |  |  |  |
| Ibáñez 2000 | 62 |  | 100 | 8 | 30 | 1 |  |  |  |  |  |  |
| Ibrahim'00 2000 | 63 |  | 100 | 5 | 1882 | 130 |  | 19 |  |  |  |  |
| Ibrahim'00 2000 | 64 |  | 69 | 11 | 4913 |  |  |  |  | 22 |  | 41 |
| Ibrahim'01 2001 | 65 |  | 56 | 9 | 880 | 49 |  |  |  |  |  |  |
| Jacobs 1990 | 66 |  | 100 | 15 | 24 | 4 |  |  |  | 1 |  |  |
| Jaillette 2011 | 67 |  | 100 | 15 | 439 | 59 |  |  |  |  |  |  |
| Jensen_HE 2015 | 68 |  | 66 | 6 | 604 |  |  |  |  |  |  | 24 |
| Jensen_SOC 2015 | 68 |  | 67 | 5 | 596 |  |  |  |  |  |  | 13 |
| Jimenez 1989 | 69 |  | 100 | 10 | 77 | 7 |  |  |  |  |  |  |
| Kautzky 2014 | 70 |  | 37 | 17 | 35 |  |  | 1 |  |  |  | 0 |
| Kautzky 2014 | 70 | crf | 57 | 24 | 30 |  |  | 1 |  |  |  | 2 |
| Ko 2013 | 71 |  | 100 | 23 | 1453 |  |  |  |  | 21 |  |  |

**Table S1 (continued): Observational studies (Benchmark groups)**

| Author | Ref | Notes | MV | LOS | Patients | VAP | | |  | Blood stream infection | | |
| --- | --- | --- | --- | --- | --- | --- | --- | --- | --- | --- | --- | --- |
|  |  |  | % | d | n | v_ps_n |  | v_can_n |  | b_ps_n |  | b_can_n |
| Kollef' 93 1993 | 72 |  | 100 | 7 | 277 | 4 |  |  |  |  |  |  |
| Kollef '95 1995 | 73 |  | 100 |  | 300 | 23 |  |  |  |  |  |  |
| Kollef '95 1995 | 74 |  | 100 | 16 | 314 | 16 |  |  |  |  |  |  |
| Kollef '97 1997 | 75 |  | 100 | 8 | 521 | 15 |  |  |  |  |  |  |
| Kollef '97_pre 1997 | 76 | C | 90 | 4 | 353 | 7 |  | 1 |  | 2 |  | 5 |
| Kollef '97_post 1997 | 76 | C I | 90 | 4 | 327 | 3 |  | 0 |  | 1 |  | 3 |
| Kollef'14_Europe 2014 | 77 |  | 100 | 11 | 495 | 24 |  |  |  |  |  |  |
| Kollef'14_USA 2014 | 77 |  | 100 | 11 | 502 | 17 |  |  |  |  |  |  |
| Koss– N 2001 | 78 |  | 100 | 14 | 87 | 4 |  | 10 |  |  |  |  |
| Koss– P 2001 | 78 | I | 100 | 11 | 66 | 9 |  | 3 |  |  |  |  |
| Kunac 2014 | 79 |  | 100 |  | 716 | 23 |  |  |  | 2 |  |  |
| Lambert 2011 | 80 |  |  | 5 | 119699 |  |  |  |  | 389 |  |  |
| Laupland 2002 | 81 |  |  | 5 | 1017 |  |  |  |  | 2 |  | 3 |
| Laupland 2004 | 82 |  | 84 | 5 | 4473 |  |  |  |  | 11 |  | 19 |
| León 2006 | 83 | crf | 95 | 21 | 1699 |  |  |  |  |  |  | 58 |
| León 2009 | 84 | crf | 91 | 17 | 1107 |  |  |  |  |  |  | 37 |
| León 2016 | 85 | crf | 84 | 15 | 233 |  |  |  |  |  |  | 11 |
| Lepelletier 2010 | 86 | T | 100 | 18 | 161 | 10 |  |  |  |  |  |  |
| Li 2016 | 87 |  | 17 | 12 | 29 |  |  | 1 |  |  |  | 0 |
| Li 2016 | 87 | crf | 40 | 25 | 82 |  |  | 10 |  |  |  | 3 |
| Luna 2003 | 88 |  | 100 | 8 | 427 | 13 |  | 2 |  |  |  |  |
| Luyt 2005 | 89 |  | 100 |  | 290 | 11 |  |  |  |  |  |  |
| Magnason 2008 | 90 |  | 100 | 8 | 280 | 5 |  | 0 |  | 0 |  | 5 |
| Mahul 1992 | 91 |  | 100 | 22 | 145 | 8 |  | 3 |  |  |  |  |
| Makris 2011 | 92 | I | 100 | 22 | 152 | 7 |  |  |  |  |  |  |
| Markowicz 2000 | 93 |  | 100 | 10 | 744 | 58 |  |  |  |  |  |  |
| Memish 2000 | 94 |  | 100 | 11 | 202 | 21 |  | 4 |  |  |  |  |
| Michel 2005 | 95 |  | 100 | 11 | 299 | 12 |  |  |  |  |  |  |
| Mitsogianni 2011 | 96 |  |  | 16 | 124 |  |  |  |  | 1 |  | 1 |
| Mitsogianni 2010 | 96 |  |  | 16 | 143 |  |  |  |  | 2 |  | 0 |
| Moine 2002 | 97 |  | 80 | 14 | 764 | 27 |  | 2 |  |  |  |  |
| Montecalvo_J 1992 | 98 |  | 100 | 10 | 38 |  |  |  |  | 1 |  | 1 |
| Myny 2005 | 99 |  | 100 | 4 | 385 | 28 |  |  |  |  |  |  |
| Nguile-Makao 2010 | 100 |  | 100 | 7 | 2873 | 130 |  |  |  |  |  |  |
| Nielsen 1992 | 101 |  | 100 | 5 | 242 | 3 |  |  |  |  |  |  |
| Nseir 2005 | 102 |  | 100 | 10 | 1241 | 32 |  |  |  |  |  |  |
| Nseir 2007 | 103 | crf | 100 | 24 | 102 |  |  |  |  |  |  | 3 |
| Orsi 2007 | 104 |  | 98 | 36 | 1741 |  |  |  |  | 29 |  | 10 |
| Orsi 2012 | 104 |  | 100 | 34 | 1165 |  |  |  |  | 12 |  | 4 |

**Table S1 (continued): Observational studies (Benchmark groups)**

| Author | Ref | Notes | MV | LOS | Patients | VAP | | |  | Blood stream infection | | |
| --- | --- | --- | --- | --- | --- | --- | --- | --- | --- | --- | --- | --- |
|  |  |  | % | d | n | v_ps_n |  | v_can_n |  | b_ps_n |  | b_can_n |
| Osmon 2003 | 105 |  | 72 | 8 | 893 |  |  |  |  | 9 |  | 26 |
| Outcomerea 2019 | 106 |  | 100 | 8 | 7735 | 454 |  |  |  |  |  |  |
| Papazian 1996 | 107 |  | 100 | 10 | 586 | 26 |  |  |  |  |  |  |
| Petri 1997 | 108 | crf | 95 | 11 | 409 |  |  |  |  |  |  | 3 |
| Potgieter 1987 | 109 |  | 78 | 9 | 250 | 26 |  | 5 |  |  |  |  |
| Prowle 2011 | 110 |  | 69 | 6 | 6339 |  |  |  |  |  |  | 51 |
| Rello'91 1991 | 111 |  | 100 | 8 | 264 | 14 |  | 1 |  |  |  |  |
| Rello'92 1992 | 112 | T | 80 | 8 | 208 | 10 |  |  |  |  |  |  |
| Rello'92 1992 | 113 |  | 67 | 9 | 161 | 4 |  |  |  |  |  |  |
| Rello'94 1994 | 114 |  | 72 |  | 1650 |  |  |  |  | 16 |  | 4 |
| Rello'02 2002 | 115 |  | 100 | 8 | 9080 | 119 |  |  |  |  |  |  |
| Rello'03 2003 | 116 |  | 100 | 20 | 99 | 8 |  | 0 |  |  |  |  |
| Resende 2013 | 117 |  | 100 | 22 | 126 | 11 |  | 0 |  |  |  |  |
| Reusser 1989 | 118 |  | 100 | 7 | 40 | 2 |  |  |  | 0 |  |  |
| Rincón-Ferrari 2004 | 119 | T | 100 | 10 | 310 | 6 |  |  |  |  |  |  |
| Rodrigues 2009 | 120 |  | 100 | 10 | 233 | 10 |  | 2 |  |  |  |  |
| Rodriguez 1991 | 121 | T | 100 | 14 | 294 | 31 |  |  |  |  |  |  |
| Ruiz-Santana 1987 | 122 |  | 100 | 7 | 1005 | 56 |  | 1 |  |  |  |  |
| Salata 1987 | 123 |  | 100 | 11 | 51 | 7 |  | 1 |  |  |  |  |
| Shahin 2013 | 124 |  | 100 | 10 | 267 | 4 |  | 3 |  |  |  |  |
| Sofianou 2000 | 125 |  | 100 | 36 | 198 | 19 |  |  |  |  |  |  |
| Stéphan 2006 | 126 | T | 100 | 16 | 175 | 14 |  |  |  |  |  |  |
| Tan 2016 | 127 | crf | 100 | 16 | 264 | 21 |  |  |  |  |  |  |
| Tan 2016 | 127 |  | 100 | 13 | 354 | 28 |  |  |  |  |  |  |
| Tejada-Artigas 2001 | 128 | T | 100 | 12 | 103 | 5 |  |  |  |  |  |  |
| Thompson 2008 | 129 |  |  | 6 | 4270 |  |  |  |  | 13 |  | 24 |
| Timsit 1996 | 130 |  | 100 | 19 | 387 | 11 |  |  |  |  |  |  |
| Torres 1990 | 131 |  | 100 | 3 | 322 | 5 |  |  |  |  |  |  |
| Trouillet 1998 | 132 |  | 100 |  | 498 | 39 |  |  |  |  |  |  |
| Urli 2002 | 133 |  | 95 | 21 | 178 | 27 |  | 1 |  | 3 |  | 4 |
| Valles 2007 | 134 |  | 100 | 22 | 60 | 15 |  |  |  |  |  |  |
| Vanhems 2011 | 135 |  | 100 | 6 | 3387 | 24 |  |  |  |  |  |  |
| Verhamme 2007 | 136 |  | 84 | 8 | 4000 | 54 |  | 6 |  |  |  |  |
| Violan 1998 | 137 |  | 100 | 16 | 314 | 25 |  |  |  |  |  |  |
| Warren 2001 | 138 |  | 28 | 4 | 3163 |  |  |  |  | 3 |  | 4 |
| Woske 2001 | 139 |  | 100 | 19 | 103 | 8 |  |  |  |  |  |  |
| Xie 2011 | 140 |  | 100 | 7 | 4155 | 169 |  | 88 |  |  |  |  |
| Xie 2020 | 141 |  | 67 | 25 | 8474 |  |  |  |  | 29 |  | 45 |
| Zahar 2009 | 142 |  | 100 | 10 | 1233 | 62 |  |  |  |  |  |  |

Table S1 footnotes

Notes: T = Data originating from a study for which the majority of ICU admission were for trauma; C = cardio-thoracic ICU; I = Infection control intervention to entire ICU; crf = group wide candidemia risk factor

MV = percentage of group receiving mechanical ventilation; NS – Not stated; LOS is mean or median length of ICU stay; ICU-LOS is the ICU length of stay.

v_ps_n is the count of *Pseudomonas* VAP; v_can_n is the count of *Candida* isolates from patients with VAP.

b_ps_n is the count of *Pseudomonas* bacteremia; b_can_n is the count of Candidemia;

Several (n = 43) of these studies were cited in the following source systematic reviews.

- Melsen WG, Rovers MM, Bonten MJM: Ventilator-associated pneumonia and mortality: A systematic review of observational studies. *Crit Care Med* 2009, 37:2709–2718.
- Safdar N, Dezfulian C, Collard HR, Saint S: Clinical and economic consequences of ventilator-associated pneumonia: a systematic review. *Crit Care Med* 2005, 33:2184–93.
- Agrafiotis M, Siempos II, Ntaidou TK, Falagas ME. Attributable mortality of ventilator-associated pneumonia: a meta-analysis. *Intern J Tub Lung Dis.* 2011;15(9):1154-1163.

**Table S2: Groups of non decontamination studies ^a^**

| Author |  | Ref | Notes | MV | LOS | Patients | VAP | | |  | Blood stream infection | | |
| --- | --- | --- | --- | --- | --- | --- | --- | --- | --- | --- | --- | --- | --- |
|  |  |  |  | % | d | n | v_ps_n |  | v_can_n |  | b_ps_n |  | b_can_n |
| Acosta-escribano 2010 |  | 143 | T | 100 | 18 | 54 | 4 |  | 1 |  |  |  |  |
| Acosta-escribano 2010 |  | 143 | T | 100 | 16 | 50 | 3 |  | 0 |  |  |  |  |
| Bonten 1995 |  | 144 |  | 100 | 17 | 67 | 11 |  |  |  |  |  | 0 |
| Bonten 1995 |  | 144 |  | 100 | 19 | 74 | 7 |  |  |  |  |  | 0 |
| Cook 1998 |  | 145 |  | 100 | 9 | 596 | 20 |  | 19 |  |  |  |  |
| Cook 1998 |  | 145 |  | 100 | 9 | 604 | 21 |  | 11 |  |  |  |  |
| Daumal 1999 |  | 146 |  | 100 | 6 | 174 | 11 |  | 2 |  |  |  |  |
| Daumal 1999 |  | 146 |  | 100 | 7 | 187 | 12 |  | 6 |  |  |  |  |
| Djedaini 1995 |  | 147 |  | 100 | 10 | 61 | 0 |  | 0 |  |  |  |  |
| Djedaini 1995 |  | 147 |  | 100 | 9 | 68 | 3 |  | 1 |  |  |  |  |
| Drakulovic 1999 |  | 148 |  | 100 | 10 | 47 | 3 |  | 0 |  |  |  |  |
| Drakulovic 1999 |  | 148 |  | 100 | 9 | 39 | 1 |  | 0 |  |  |  |  |
| Dreyfuss 1991 |  | 149 |  | 100 | 10 | 35 | 3 |  |  |  |  |  |  |
| Dreyfuss 1991 |  | 149 |  | 100 | 13 | 28 | 1 |  |  |  |  |  |  |
| Dreyfuss 1995 |  | 150 |  | 100 | 10 | 70 | 1 |  |  |  |  |  |  |
| Dreyfuss 1995 |  | 150 |  | 100 | 13 | 61 | 0 |  |  |  |  |  |  |
| Driks 1987 |  | 151 |  | 100 | 14 | 69 | 5 |  | 0 |  |  |  |  |
| Driks 1987 |  | 151 |  | 100 | 11 | 61 | 1 |  | 0 |  |  |  |  |
| Forestier 2008 |  | 152 | T | 100 | 13 | 106 | 8 |  |  |  |  |  |  |
| Forestier 2008 |  | 152 | T | 100 | 13 | 102 | 3 |  |  |  |  |  |  |
| Heyland 1999 |  | 153 |  | 100 | 12 | 46 | 0 |  | 0 |  |  |  |  |
| Heyland 1999 |  | 153 |  | 100 | 13 | 49 | 0 |  | 0 |  |  |  |  |
| Holzapfel_C 1999 |  | 154 |  | 100 | 15 | 200 | 6 |  | 3 |  | 0 |  | 1 |
| Holzapfel_I 1999 |  | 154 |  | 100 | 17 | 199 | 10 |  | 1 |  | 2 |  | 1 |
| Kappstein 1991 |  | 155 | T | 100 | 5 | 55 |  |  | 7 |  |  |  |  |
| Kappstein 1991 |  | 155 | T | 100 | 5 | 49 |  |  | 3 |  |  |  |  |
| Kirschenbaum 2002 |  | 156 |  | 100 | 20 | 20 | 5 |  |  |  |  |  |  |
| Kirschenbaum 2002 |  | 156 |  | 100 | 21 | 17 | 1 |  |  |  |  |  |  |
| Kirton 1997 |  | 157 | T | 100 |  | 140 | 6 |  |  |  |  |  |  |
| Kirton 1997 |  | 157 | T | 100 |  | 140 | 6 |  |  |  |  |  |  |
| Knight 2009 |  | 158 |  | 100 | 7 | 129 | 1 |  | 0 |  |  |  |  |
| Knight 2009 |  | 158 |  | 100 | 6 | 130 | 0 |  | 0 |  |  |  |  |
| Kollef 2008 |  | 159 |  | 100 | 4 | 743 | 11 |  | 7 |  |  |  |  |
| Kollef_silverETT  2008 |  | 159 |  | 100 | 4 | 766 | 8 |  | 5 |  |  |  |  |
| Lacherade 2005 |  | 160 |  | 100 | 25 | 184 | 14 |  | 2 |  |  |  |  |
| Lacherade 2005 |  | 160 |  | 100 | 21 | 185 | 9 |  | 0 |  |  |  |  |
| Lacherade 2010 | 161 |  | 100 | 11 | 164 | 16 |  |  |  |  |  |  |  |
| Lacherade 2010 | 161 |  | 100 | 11 | 169 | 9 |  |  |  |  |  |  |  |

**Table S2 (continued): Groups of non decontamination studies ^a^**

| Author | Ref | Notes | MV | LOS | Patients | VAP | | |  | Blood stream infection | | |
| --- | --- | --- | --- | --- | --- | --- | --- | --- | --- | --- | --- | --- |
|  |  |  | % | d | n | v_ps_n |  | v_can_n |  | b_ps_n |  | b_can_n |
| Lorente 2003 | 162 |  | 100 | 18 | 116 | 10 |  | 3 |  |  |  |  |
| Lorente 2003 | 162 |  | 100 | 16 | 114 | 9 |  | 4 |  |  |  |  |
| Lorente 2004 | 163 |  | 100 | 16 | 143 | 8 |  | 3 |  |  |  |  |
| Lorente 2004 | 163 |  | 100 | 20 | 161 | 9 |  | 2 |  |  |  |  |
| Lorente2006 | 164 |  | 100 | 13 | 233 | 12 |  | 1 |  |  |  |  |
| Lorente 2006 | 164 |  | 100 | 13 | 210 | 12 |  | 2 |  |  |  |  |
| Lorente'06a 2005 | 165 |  | 100 | 10 | 221 | 7 |  | 1 |  |  |  |  |
| Lorente'06a 2005 | 165 |  | 100 | 10 | 236 | 9 |  | 1 |  |  |  |  |
| Lorente 2006 | 166 |  | 100 | 21 | 51 | 5 |  | 0 |  |  |  |  |
| Lorente 2006 | 166 |  | 100 | 20 | 53 | 2 |  | 0 |  |  |  |  |
| Lorente 2007 | 167 |  | 100 | 16 | 140 | 4 |  | 0 |  |  |  |  |
| Lorente 2007 | 167 |  | 100 | 14 | 140 | 4 |  | 0 |  |  |  |  |
| Lorente 2014 | 168 |  | 100 | 16 | 150 | 6 |  |  |  |  |  |  |
| Lorente 2014 | 168 |  | 100 | 15 | 134 | 3 |  |  |  |  |  |  |
| Manzano 2008 | 169 |  | 100 | 12 | 63 | 0 |  |  |  |  |  |  |
| Manzano 2008 | 169 |  | 100 | 9 | 64 | 0 |  |  |  |  |  |  |
| Martin 1993 | 170 |  | 100 | 10 | 66 | 2 |  | 0 |  |  |  |  |
| Martin 1993 | 170 |  | 100 | 10 | 65 | 0 |  | 1 |  |  |  |  |
| Morrow 2010 | 171 |  | 100 | 15 | 73 | 6 |  | 1 |  |  |  |  |
| Morrow 2010 | 171 |  | 100 | 15 | 73 | 0 |  | 0 |  |  |  |  |
| Nseir 2011 | 172 |  | 100 | 10 | 61 | 2 |  |  |  |  |  |  |
| Nseir 2011 | 172 |  | 100 | 12 | 61 | 0 |  |  |  |  |  |  |
| Pickworth 1993 | 173 | T | 100 | 7 | 44 | 1 |  | 0 |  |  |  |  |
| Pneumatikos 2006 | 174 | T | 100 | 16 | 40 | 1 |  | 0 |  |  |  |  |
| Pneumatikos 2006 | 174 | T | 100 | 15 | 39 | 0 |  | 0 |  |  |  |  |
| Prod'hom_A 1994 | 175 |  | 100 | 6 | 81 | 4 |  |  |  |  |  |  |
| Prod'hom_S 1994 | 175 |  | 100 | 5 | 83 | 1 |  |  |  |  |  |  |
| Prod'hom_R 1994 | 175 |  | 100 | 5 | 80 | 1 |  |  |  |  |  |  |
| Reigneir 2013 | 176 |  | 100 | 10 | 222 | 9 |  |  |  |  |  |  |
| Reigneir 2013 | 176 |  | 100 | 10 | 227 | 12 |  |  |  |  |  |  |
| Rumbak 2004 | 177 |  | 100 | 5 | 60 | 5 |  |  |  |  |  |  |
| Rumbak 2004 | 177 |  | 100 | 16 | 60 | 1 |  |  |  |  |  |  |
| Ryan_C 1993 |  | 178 |  | 100 | 5 | 56 | 2 |  |  |  |  |  |
| Ryan_S 1993 |  | 178 |  | 100 | 6 | 58 | 1 |  |  |  |  |  |
| Smulders 2002 |  | 179 |  | 100 | 14 | 75 | 3 |  | 1 |  |  |  |
| Smulders 2002 |  | 179 |  | 100 | 12 | 75 | 1 |  | 0 |  |  |  |
| Staudinger 2010 |  | 180 |  | 100 | 14 | 75 | 5 |  | 2 |  |  |  |
| Staudinger 2010 |  | 180 |  | 100 | 8 | 75 | 3 |  | 0 |  |  |  |
| Thomachot 1998 |  | 181 |  | 100 | 12 | 66 | 3 |  | 0 |  |  |  |
| Thomachot 1998 |  | 181 |  | 100 | 12 | 70 | 2 |  | 0 |  |  |  |

**Table S2 (continued): Groups of non decontamination studies ^a^**

| Author | Ref | Notes | MV | LOS | Patients |  | VAP | | | Blood stream infection | | |
| --- | --- | --- | --- | --- | --- | --- | --- | --- | --- | --- | --- | --- |
|  |  |  |  | % | d | n | v_ps_n |  | v_can_n | b_ps_n |  | b_can_n |
| Thomachot 1999 |  | 182 | T | 100 | 11 | 77 | 1 |  | 0 |  |  |  |
| Thomachot 1999 |  | 182 | T | 100 | 12 | 63 | 2 |  | 0 |  |  |  |
| Thomachot 2002 |  | 183 | T | 100 | 9 | 84 | 2 |  |  |  |  |  |
| Thomachot 2002 |  | 183 | T | 100 | 9 | 71 | 0 |  |  |  |  |  |
| Valencia 2007 |  | 184 |  | 100 | 13 | 69 | 1 |  |  |  |  |  |
| Valencia 2007 |  | 184 |  | 100 | 13 | 73 | 3 |  |  |  |  |  |
| Walaszek 2017 |  | 185 |  | 100 | 5 | 804 | 5 |  | 5 |  |  |  |
| Walaszek 2017 |  | 185 |  | 100 | 5 | 1003 | 10 |  | 1 |  |  |  |
| Zeng 2016 |  | 186 |  | 100 | 22 | 117 | 19 |  | 4 |  |  |  |
| Zeng 2016 |  | 186 |  | 100 | 18 | 118 | 13 |  | 2 |  |  |  |
|  |  |  |  |  |  |  |  |  |  |  |  |  |

Table S2 footnotes

Notes; T = Data originating from a study for which the majority of ICU admission were for trauma; C = cardio-thoracic ICU; crf = group wide candidemia risk factor;

MV = percentage of group receiving mechanical ventilation; NS – Not stated; LOS is mean or median length of ICU stay; ICU-LOS is the ICU length of stay.

v_ps_n is the count of *Pseudomonas* VAP; v_can_n is the count of *Candida* isolates from patients with VAP.

b_ps_n is the count of *Pseudomonas* bacteremia; b_can_n is the count of Candidemia;

Several (n = 47) of these studies were cited in the following source systematic reviews.

- Messori A, Trippoli S, Vaiani M, Gorini M, Corrado A: Bleeding and pneumonia in intensive care patients given ranitidine and sucralfate for prevention of stress ulcer: meta-analysis of randomised controlled trials. *BMJ* 2000, 321:1103–1106.
- Huang J, Cao Y, Liao C, Wu L, Gao F: Effect of histamine-2-receptor antagonists versus sucralfate on stress ulcer prophylaxis in mechanically ventilated patients: a meta-analysis of 10 randomized controlled trials. *Crit Care* 2010, 14:R194.
- Alhazzani W, Almasoud A, Jaeschke R, Lo BW, Sindi A, Altayyar S, Fox-Robichaud A: Small bowel feeding and risk of pneumonia in adult critically ill patients: a systematic review and meta-analysis of randomized trials. *Crit Care* 2013, 17:R127.
- Melsen WG, Rovers MM, Bonten MJM: Ventilator-associated pneumonia and mortality: A systematic review of observational studies. *Crit Care Med* 2009, 37:2709–2718.
- Safdar N, Dezfulian C, Collard HR, Saint S: Clinical and economic consequences of ventilator-associated pneumonia: a systematic review. *Crit Care Med* 2005, 33:2184–93.
- Han J, Liu Y. Effect of ventilator circuit changes on ventilator-associated pneumonia: a systematic review and meta-analysis. *Respiratory care*, 2010; 55: 467-474.
- Subirana M, Solà I, Benito S: Closed tracheal suction systems versus open tracheal suction systems for mechanically ventilated adult patients. *Cochrane Database Syst Rev* 2007, 4: CD004581;
- Siempos II, Vardakas KZ, Kopterides P, Falagas ME. Impact of passive humidification on clinical outcomes of mechanically ventilated patients: A meta-analysis of randomized controlled trials. *Crit Care Med* 2007; 35: 2843-51;
- Muscedere J, Rewa O, McKechnie K, Jiang X, Laporta D, Heyland DK. Subglottic secretion drainage for the prevention of ventilator-associated pneumonia: a systematic review and meta-analysis. *Crit Care Med* 2011; 39:1985–1991.
- Delaney A, Gray H, Laupland KB, Zuege DJ. Kinetic bed therapy to prevent nosocomial pneumonia in mechanically ventilated patients: a systematic review and meta-analysis. *Crit Care* 2006; 10:R70;
- Sud S, Friedrich JO, Taccone P, Polli F, Adhikari NK, Latini R, Gattinoni L. Prone ventilation reduces mortality in patients with acute respiratory failure and severe hypoxemia: systematic review and meta-analysis. *Inten Care Med* 2010; 36(4); 585-599.
- Siempos II, Vardakas KZ, Falagas ME. Closed tracheal suction systems for prevention of ventilator-associated pneumonia. *Brit J Anaesthesia*, 2008; 100(3): 299-306.

**Table S3: Groups of anti-septic studies ^a^**

| Author  & regimen | Ref | Notes | MV | LOS | Patients | VAP | | |  | Blood stream infection | | |
| --- | --- | --- | --- | --- | --- | --- | --- | --- | --- | --- | --- | --- |
|  |  |  | % | d | n | v_ps_n |  | v_can_n |  | b_ps_n |  | b_can_n |
| Bellissimo-Rodrigues  2014 | 187 |  | 76 | 11 | 127 |  |  | 1 |  |  |  |  |
| Bellissimo-Rodrigues  2014_Chlx | 187 |  | 77 | 11 | 127 |  |  | 0 |  |  |  |  |
| Bleasdale 2007 | 188 |  | 35 | 3 | 445 |  |  |  |  | 0 |  | 2 |
| Bleasdale 2007_Chlx | 188 |  | 36 | 3 | 391 |  |  |  |  | 0 |  | 2 |
| Cabov 2010 | 189 |  | 57 | 6 | 30 | 4 |  |  |  | 0 |  |  |
| Cabov 2010_Chlx | 189 |  | 77 | 6 | 30 | 0 |  |  |  | 0 |  |  |
| Caruso 2009 | 190 |  | 100 | 17 | 132 | 9 |  | 3 |  |  |  |  |
| Caruso 2009_Saline | 190 |  | 100 | 17 | 130 | 7 |  | 0 |  |  |  |  |
| Climo_NC 2013 | 191 |  |  | 6 | 1398 |  |  |  |  | 2 |  | 16 |
| Climo 2013_Chlx | 191 |  |  | 6 | 1410 |  |  |  |  | 4 |  | 7 |
| Fourrier 2000 | 192 |  | 100 | 24 | 30 | 4 |  | 1 |  | 0 |  | 0 |
| Fourrier 2000_Chlx | 192 |  | 100 | 18 | 30 | 1 |  | 0 |  | 0 |  | 0 |
| Fourrier 2005 | 193 |  | 100 | 13 | 114 | 5 |  | 0 |  | 0 |  | 0 |
| Fourrier 2005_Chlx | 193 |  | 100 | 14 | 114 | 6 |  | 0 |  | 1 |  | 0 |
| Huang_2pre 2013 | 58 |  |  | 3 | 15218 |  |  |  |  | 8 |  | 56 |
| Huang_3pre 2013 | 58 |  |  | 3 | 17356 |  |  |  |  | 11 |  | 59 |
| Huang_3UD 2013_Chlx | 58 |  |  | 3 | 26024 |  |  |  |  | 14 |  | 62 |
| Huang_2TD 2013_Chlx | 58 |  |  | 3 | 24752 |  |  |  |  | 13 |  | 63 |
| Koeman 2006 | 194 |  | 100 | 13 | 130 | 4 |  | 1 |  |  |  |  |
| Koeman 2006_Chlx | 194 |  | 100 | 14 | 127 | 0 |  | 3 |  |  |  |  |
| Koeman 2006_ChlxC | 194 |  | 100 | 13 | 128 | 2 |  | 4 |  |  |  |  |
| Kollef 2006 | 195 |  | 100 | 14 | 347 | 9 |  | 6 |  |  |  |  |
| Kollef 2006_Iseganin | 195 |  | 100 | 14 | 362 | 8 |  | 0 |  |  |  |  |
| Lorente 2012 | 196 |  | 100 | 9 | 219 | 5 |  | 0 |  |  |  |  |
| Lorente 2012_Chlx | 196 |  | 100 | 10 | 217 | 5 |  | 0 |  |  |  |  |
| Milstone 2013 | 197 | P |  | 3 | 1326 |  |  |  |  | 1 |  | 6 |
| Milstone 2013_Chlx | 197 | P |  | 3 | 667 |  |  |  |  | 1 |  | 3 |
| Mori H 2006 | 198 |  | 100 | 7 | 414 | 9 |  | 2 |  |  |  |  |
| Mori 2006-PVI | 198 |  | 100 | 8 | 1248 | 10 |  | 1 |  |  |  |  |
| Noto_pre 2015 | 199 |  |  | 3 | 4852 |  |  |  |  | 2 |  | 6 |
| Noto_post 2015_Chlx | 199 |  |  | 3 | 4488 |  |  |  |  | 4 |  | 2 |
| Seguin – SC 2006 | 200 | T | 100 | 14 | 31 | 1 |  |  |  |  |  |  |
| Seguin – CC 2006 | 200 | T | 100 | 19 | 31 | 0 |  |  |  |  |  |  |
| Seguin 2006-PVI | 200 | T | 100 | 15 | 36 | 0 |  |  |  |  |  |  |
|  |  |  |  |  |  |  |  |  |  |  |  |  |

**Table S3 (continued): Groups of anti-septic studies ^a^**

| Author  & regimen | Ref | Notes | MV | LOS | Patients | VAP | | |  | Blood stream infection | | |
| --- | --- | --- | --- | --- | --- | --- | --- | --- | --- | --- | --- | --- |
|  |  |  | % | d | n | v_ps_n |  | v_can_n |  | b_ps_n |  | b_can_n |
| Seguin 2014 | 201 | T | 100 | 16 | 72 | 1 |  |  |  |  |  |  |
| Seguin 2014-PVI | 201 | T | 100 | 15 | 78 | 3 |  |  |  |  |  |  |
| Swan 2016 | 202 |  | 57 | 7 | 164 | 1 |  | 1 |  | 0 |  |  |
| Swan 2016_Chlx | 202 |  | 69 | 7 | 161 | 1 |  | 0 |  | 0 |  |  |
| Wittekamp 2018 | 203 |  | 100 | 10 | 2251 |  |  |  |  | 21 |  | 15 |
| Wittekamp 2018_Chlx | 203 |  | 100 | 10 | 2108 |  |  |  |  | 16 |  | 22 |
|  |  |  |  |  |  |  |  |  |  |  |  |  |

Table S3: Footnotes

Notes; T = Data originating from a study for which the majority of ICU admission were for trauma; P = paediatric ICU; I = Infection control intervention to entire ICU; crf = group wide candidemia risk factor;

MV = percentage of group receiving mechanical ventilation; NS – Not stated; LOS is mean or median length of ICU stay; ICU-LOS is the ICU length of stay.

v_ps_n is the count of *Pseudomonas* VAP; v_can_n is the count of *Candida* isolates from patients with VAP.

b_ps_n is the count of *Pseudomonas* bacteremia; b_can_n is the count of Candidemia;

Several (n = 5) of these studies were cited in the following source meta-analyses.

- Chan EY, Ruest A, Meade MO, Cook DJ: Oral decontamination for prevention of pneumonia in mechanically ventilated adults: systematic review and meta-analysis. *BMJ* 2007, 334:889–900.
- Labeau SO, Van de Vyver K, Brusselaers N, Vogelaers D, Blot SI: Prevention of ventilator-associated pneumonia with oral antiseptics: a systematic review and meta-analysis. *Lancet Infect Dis* 2011, 11:845-854.
- Pileggi C, Bianco A, Flotta D, *et al*. Prevention of ventilator-associated pneumonia, mortality and all intensive care unit acquired infections by topically applied antimicrobial or antiseptic agents: a meta-analysis of randomized controlled trials in intensive care units. *Crit Care.* 2011; **15**: R155.
- Price R, MacLennan G, Glen J. Selective digestive or oropharyngeal decontamination and topical oropharyngeal chlorhexidine for prevention of death in general intensive care: systematic review and network meta-analysis. *BMJ*. 2014; **348**: g2197.
- Klompas M, Speck K, Howell MD, *et al*. Reappraisal of routine oral care with chlorhexidine gluconate for patients receiving mechanical ventilation: systematic review and meta-analysis. *JAMA Intern Med.* 2014; **174**: 751-61.

Intervention regimens abbreviations

Chlx = chlorhexidine; Chlx BW = chlorhexidine body wash; ChC = chlorhexidine and colisitn; TD = targetted decolonization; UD = universal decolonization; PVI = povidone iodine; CC = concurrent control; SC = saline control; iseganan, is a synthetic variant of a porcine protegrin, which is a natural antibiotic peptide released by neutrophils in response to invasion by microbes [Kollef 2006].

**Table S4: Groups of antibiotic-based prophylaxis (=TAP±PPAP±antifungal) studies ^a^**

| Author  & regimen | Ref | Notes | MV | LOS | Patients | VAP | | |  | Blood stream infection | | |
| --- | --- | --- | --- | --- | --- | --- | --- | --- | --- | --- | --- | --- |
|  |  |  | % | d | n | v_ps_n |  | v_can_n |  | b_ps_n |  | b_can_n |
| Groups from  NCC studies |  |  |  |  |  |  |  |  |  |  |  |  |
| Bergmans NC 2001 | 204 |  | 100 | 12 | 61 | 5 |  | 1 |  |  |  |  |
| Bergmans CC 2001 | 204 |  | 100 | 15 | 78 | 8 |  | 3 |  |  |  |  |
| Bergmans 2001_PGV | 204 |  | 100 | 13 | 87 | 3 |  | 1 |  |  |  |  |
| Bonten NC 1994 | 205 |  | 91 | 16 | 54 | 4 |  |  |  |  |  |  |
| Bonten CC 1994 | 205 |  | 86 | 9 | 21 | 0 |  |  |  |  |  |  |
| Bonten 1994_PTA | 205 |  | 100 | 13 | 22 | 0 |  |  |  |  |  |  |
| Camus NC 2014 | 206 |  | 28 | 4 | 925 | 16 |  | 3 |  | 0 |  | 2 |
| Camus 2014_PT | 206 |  | 28 | 4 | 1022 | 5 |  | 1 |  | 3 |  | 1 |
| De La Court 2021_PTA | 207 |  |  |  | 722 |  |  |  |  | 3 |  | 4 |
| De La Court 2021_PTA | 207 |  |  |  | 1236 |  |  |  |  | 4 |  | 11 |
| de Smet 2009 | 208 |  | 88 | 9 | 1990 |  |  |  |  | 36* |  | 16 |
| de Smet SDD 2009_PTA | 208 |  | 93 | 9 | 2045 |  |  |  |  | 17* |  | 8 |
| de Smet SOD 2009_PTA | 208 |  | 94 | 9 | 1904 |  |  |  |  | 16* |  | 14 |
| Frencken 2018_PTA | 209 |  | 100 | 7 | 1874 |  |  |  |  | 15 |  |  |
| Godard 1990 | 210 |  | 80 | 13 | 84 | 5 |  |  |  |  |  | 2 |
| Godard 1990_PT | 210 |  | 81 | 11 | 97 | 0 |  |  |  |  |  | 0 |
| Gorensek 1993 | 211 | crf |  | 15 | 34 |  |  |  |  | 2 |  | 1 |
| Gorensek 1993_NoNy | 211 | crf |  | 15 | 17 |  |  |  |  | 1 |  | 0 |
| Hartenauer 1990 | 212 |  | 100 | 14 | 101 | 20 |  | 0 |  | 1 |  | 0 |
| Hartenauer 1990_PTA | 212 |  | 100 | 13 | 99 | 0 |  | 1 |  | 1 |  | 3 |
| Hjortrup 1997_CefTNy | 213 | crf | 100 |  | 150 |  |  | 11 |  | 1 |  | 4 |
| Konrad 1989 | 214 |  | 100 |  | 83 | 9 |  |  |  |  |  |  |
| Konrad 1989_PTA | 214 |  | 100 |  | 82 | 2 |  |  |  |  |  |  |
| Landelle 2018 | 215 |  | 100 | 9 | 291 | 9 |  |  |  |  |  |  |
| Landelle 2018_PTNy | 215 |  | 100 | 8 | 413 | 12 |  |  |  |  |  |  |
| Landelle 2018_PTNy | 215 |  | 100 | 9 | 356 | 2 |  |  |  |  |  |  |
| Ledingham 1988 | 216 |  | 60 | 5 | 161 | 3 |  |  |  |  |  |  |
| Ledingham 1988_PTA | 216 |  | 60 | 5 | 163 | 2 |  |  |  |  |  |  |
| Leone 2002_PTA | 217 | T | 100 | 12 | 324 | 3 |  |  |  |  |  |  |
| Mathieu 2day 2020_PTA | 218 | T | 100 |  | 199 |  |  |  |  |  |  | 0 |
| Mathieu 3day 2020_PTA | 218 | T | 100 |  | 248 |  |  |  |  |  |  | 1 |
|  |  |  |  |  |  |  |  |  |  |  |  |  |

**Table S4 (continued): Groups of antibiotic-based prophylaxis (=TAP±PPAP±antifungal) studies ^a^**

| Author  & regimen | Ref | Notes | MV | LOS | Patients | VAP | | |  | Blood stream infection | | |
| --- | --- | --- | --- | --- | --- | --- | --- | --- | --- | --- | --- | --- |
|  |  |  | % | d | n | v_ps_n |  | v_can_n |  | b_ps_n |  | b_can_n |
| Groups from  NCC studies |  |  |  |  |  |  |  |  |  |  |  |  |
| Nardi 2001_PTAM | 219 | T | 100 | 11 | 119 | 3 |  | 0 |  |  |  |  |
| Nardi 2001_PTA | 219 | T | 100 | 12 | 104 | 4 |  | 1 |  |  |  |  |
| Ong 2015_PTA | 220 |  | 87 | 10 | 3080 |  |  |  |  | 10 |  |  |
| Oostdijk 2011 | 221 |  | 88 | 9 | 1945 |  |  |  |  | 25* |  |  |
| Oostdijk 2011_PTA | 221 |  | 94 | 9 | 2166 |  |  |  |  | 16* |  |  |
| Oostdijk_2011 PTA | 221 |  | 93 | 9 | 2667 |  |  |  |  | 14* |  |  |
| Oostdijk SDD 2014_PTA | 222 |  | 51 | 6 | 5483 |  |  |  |  | 20 |  |  |
| Oostdijk SOD 2014_PTA | 222 |  | 52 | 6 | 5508 |  |  |  |  | 23 |  |  |
| Rouby 1994_P | 223 |  | 100 | 12 | 347 | 12 |  |  |  |  |  |  |
| Rouby 1994_E | 223 |  | 100 | 18 | 251 | 26 |  |  |  |  |  |  |
| Silvestri 1999_PTA | 224 | e | 100 | 9 | 117 | 2 |  | 0 |  | 0 |  |  |
| Steffen 1994_PTNy | 225 | crf | 100 | 14 | 127 |  |  |  |  | 1 |  |  |
| Stoutenbeek  1984 | 226 | T | 100 | 14 | 59 | 5 |  | 0 |  | 1 |  |  |
| Stoutenbeek  1984_PTA | 226 | T | 100 | 11 | 63 | 3 |  | 0 |  | 0 |  |  |
| Stoutenbeek  1987 | 227 | T | 100 | 14 | 59 | 5 |  | 0 |  |  |  |  |
| Stoutenbeek  SDD 1987_PTA | 227 | T | 100 | 11 | 63 | 0 |  | 0 |  | 0 |  |  |
| Stoutenbeek  ED 1987_PTA | 227 | T | 100 | 18 | 42 | 1 |  | 0 |  | 1 |  |  |
| Valles 2013 | 228 |  | 100 | 15 | 58 | 2 |  |  |  |  |  |  |
| Valles 2013 | 228 |  | 100 | 10 | 71 | 1 |  |  |  |  |  |  |
| Veelo 2008_PTA | 229 |  | 100 | 17 | 231 | 2 |  |  |  |  |  |  |
| Winter CC 1992 | 230 |  | 92 | 8 | 92 | 8 |  | 0 |  |  |  |  |
| Winter NC 1992 | 230 |  | 92 | 7 | 84 | 2 |  | 0 |  |  |  |  |
| Winter 1992_PTA | 230 |  | 92 | 6 | 91 | 3 |  | 0 |  |  |  |  |
| Wittekamp 2018_ PTNy | 203 |  | 100 | 10 | 2224 |  |  |  |  | 15 |  | 23 |
| Wittekamp 2018_PTNy | 203 |  | 100 | 11 | 2082 |  |  |  |  | 9 |  | 18 |
|  |  |  |  |  |  |  |  |  |  |  |  |  |

**Table S4 (continued): Groups of antibiotic-based prophylaxis (=TAP±PPAP±antifungal) studies ^a^**

| Author & regimen | Ref | Notes | MV | LOS | Patients | VAP | | |  | Blood stream infection | | |
| --- | --- | --- | --- | --- | --- | --- | --- | --- | --- | --- | --- | --- |
|  |  |  | % | d | n | v_ps_n |  | v_can_n |  | b_ps_n |  | b_can_n |
| Groups from  CC studies |  |  |  |  |  |  |  |  |  |  |  |  |
| Abele-Horn 1997 | 231 | T | 100 | 22 | 30 | 3 |  |  |  |  |  |  |
| Abele-Horn 1997_ PTA | 231 | T | 100 | 18 | 58 | 2 |  |  |  |  |  |  |
| Acquarolo 2005 | 232 |  | 100 | 13 | 19 | 2 |  | 0 |  | 0 |  |  |
| Acquarolo 2005_AmpSul | 232 |  | 100 | 13 | 19 | 2 |  | 0 |  | 0 |  |  |
| Aerdts 1991 | 233 |  | 100 | 28 | 39 | 10 |  | 2 |  |  |  | 1 |
| Aerdts 1991_ PNoA | 233 |  | 100 | 23 | 17 | 0 |  | 0 |  |  |  | 0 |
| Bion 1991 | 234 | crf | 50 | 2 | 31 | 3 |  | 2 |  | 0 |  | 0 |
| Bion 1991_ PTA | 234 | crf | 50 | 2 | 21 | 0 |  | 3 |  | 2 |  | 0 |
| Blair 1991 | 235 |  | 93 | 8 | 130 | 9 |  | 1 |  | 3 |  | 0 |
| Blair 1991_ PTA | 235 |  | 93 | 8 | 126 | 1 |  | 0 |  | 1 |  | 1 |
| Blaise 1994 | 236 | crf | 33 | 7 | 45 | 0 |  |  |  | 1 |  |  |
| Blaise 1994_O | 236 | crf | 37 | 7 | 46 | 1 |  |  |  | 1 |  |  |
| Bouza 2013 | 237 |  | 100 | 12 | 38 | 3 |  | 0 |  |  |  |  |
| Bouza 2013_Lnz_Mrp | 237 |  | 100 | 10 | 40 | 3 |  | 0 |  |  |  |  |
| Cerra 1992 | 238 | e |  | 26 | 21 | 6 |  | 10 |  | 1 |  | 4 |
| Cerra 1992_NoNy | 238 | e |  | 18 | 25 | 4 |  | 6 |  | 1 |  | 1 |
| Cockerill 1992 | 239 |  | 85 | 12 | 75 | 4 |  |  |  |  |  | 2 |
| Cockerill 1992_ PGNy | 239 |  | 85 | 10 | 75 | 1 |  |  |  |  |  | 1 |
| de la Cal 2005 | 240 | T | 80 | 34 | 54 | 7 |  | 0 |  | 7 |  | 0 |
| de la Cal 2005_ PTA | 240 | T | 74 | 31 | 53 | 2 |  | 2 |  | 9 |  | 1 |
| Ferrer 1994 | 241 |  | 100 | 14 | 41 | 4 |  | 2 |  | 0 |  | 0 |
| Ferrer 1994_ PTA | 241 |  | 100 | 15 | 39 | 1 |  | 0 |  | 1 |  | 0 |
| Flaherty 1990 | 242 |  | 40 | 4 | 56 |  |  |  |  |  |  | 0 |
| Flaherty 1990_ PGA | 242 |  | 40 | 4 | 51 |  |  |  |  |  |  | 0 |
| Garbino_EN 2004_ PNeV | 243 |  | 100 | 14 | 71 |  |  | 1 |  |  |  | 3 |
| Garbino_TPN 2004_ PNeV | 243 |  | 100 | 14 | 29 |  |  | 0 |  |  |  | 2 |
| Gaussorgues 1991 | 244 |  | 100 | 17 | 59 |  |  | 1 |  | 7 |  | 15 |
| Gaussorgues 1991_ PGA | 244 |  | 100 | 16 | 59 |  |  | 0 |  | 1 |  | 5 |
| Hellinger 2002 | 245 | crf |  | 21 | 43 | 1 |  | 0 |  |  |  | 0 |
| Hellinger 2002_ PGNy | 245 | crf |  | 21 | 37 | 0 |  | 0 |  |  |  | 0 |
| Jacobs 1992 | 246 |  | 100 | 10 | 43 | 0 |  | 0 |  | 0 |  | 0 |
| Jacobs 1992_ PTA | 246 |  | 100 | 9 | 36 | 0 |  | 0 |  | 0 |  | 0 |
| Kerver 1988 | 247 |  | 100 | 20 | 47 |  |  | 0 |  |  |  | 0 |
| Kerver 1988_ PTA | 247 |  | 100 | 17 | 49 |  |  | 0 |  |  |  | 0 |
| Korinek 1993 | 248 | T | 100 | 27 | 60 | 3 |  | 0 |  |  |  |  |
| Korinek 1993_ PTA | 248 | T | 100 | 25 | 63 | 0 |  | 0 |  |  |  |  |
| Laggner 1994 | 249 |  | 100 | 20 | 34 | 1 |  | 0 |  | 1 |  | 0 |
| Laggner 1994_ PA | 249 |  | 100 | 16 | 33 | 0 |  | 0 |  | 0 |  | 0 |

**Table S4 (continued): Groups of antibiotic-based prophylaxis (=TAP±PPAP±antifungal) studies ^a^**

| Author & regimen | Ref | Notes | MV | LOS | Patients | VAP | | |  | Blood stream infection | | |
| --- | --- | --- | --- | --- | --- | --- | --- | --- | --- | --- | --- | --- |
|  |  |  | % | d | n | v_ps_n |  | v_can_n |  | b_ps_n |  | b_can_n |
| Palomar 1997 | 250 |  | 100 | 6 | 42 | 6 |  | 0 |  |  |  | 0 |
| Palomar Ctx 1997 | 250 |  | 100 | 8 | 46 | 1 |  |  |  |  |  | 1 |
| Palomar_1 1997 | 250 |  | 100 | 11 | 41 | 1 |  | 0 |  |  |  | 0 |
| Pneumatikos 2002 | 251 | T | 100 | 23 | 30 | 1 |  | 0 |  |  |  |  |
| Pneumatikos 2002_ PTA | 251 | T | 100 | 16 | 31 | 0 |  | 0 |  |  |  |  |
| Quinio 1995 | 252 | T | 100 | 16 | 72 | 12 |  | 0 |  | 0 |  | 0 |
| Quinio 1995_ PGA | 252 | T | 100 | 16 | 76 | 5 |  | 0 |  | 0 |  | 0 |
| Rocha 1992 | 253 | T | 100 | 18 | 54 | 8 |  |  |  | 1 |  | 0 |
| Rocha 1992_ PTA | 253 | T | 100 | 19 | 47 | 1 |  |  |  | 0 |  | 0 |
| Rodríguez-Roldán  1990 | 254 |  | 100 | 12 | 15 | 5 |  |  |  |  |  |  |
| Rodríguez-Roldán  1990_ PTNeA | 254 |  | 100 | 10 | 13 | 0 |  |  |  |  |  |  |
| Rolando 1996 | 255 | crf | 73 |  | 61 | 2 | 1 | 1 |  | 0 | 1 | 1 |
| Rolando 1996_ PTA | 255 | crf | 73 |  | 47 | 1 | 2 | 1 |  | 0 | 0 | 0 |
| Rolando 1993 | 256 | crf | 75 | 8 | 31 | 2 | 1 | 3 |  |  | 3 | 0 |
| Rolando 1993_ PTAM | 256 | crf | 75 | 8 | 28 | 1 | 2 | 0 |  |  | 0 | 0 |
| Rolando_infected  1993 | 256 | crf | 75 | 8 | 21 | 0 | 0 | 1 |  |  | 0 | 0 |
| Rolando_infected  1993_ PTAM | 256 | crf | 75 | 7 | 21 | 0 | 1 | 1 |  |  | 0 | 0 |
| Sanchez-Garcia 1998 | 257 |  | 100 | 20 | 140 |  | 7 | 0 |  |  |  |  |
| Sanchez-Garcia 1998_ PTA | 257 |  | 100 | 17 | 131 |  | 5 | 0 |  |  |  |  |
| Sirvent 1997 | 258 | T | 100 | 16 | 50 | 1 | 11 | 0 |  |  |  |  |
| Sirvent 1997 | 258 | T | 100 | 13 | 50 | 3 | 3 | 0 |  |  |  |  |
| Smith 1993 | 259 | crf | 100 | 8 | 18 | 1 |  | 1 |  |  |  | 1 |
| Smith 1993_ PTA | 259 | crf | 100 | 7 | 18 | 0 |  | 0 |  |  |  | 0 |
| Stoutenbeek 2007 | 260 | T | 100 | 12 | 200 | 28 | 40 | 21 |  | 2 | 5 | 0 |
| Stoutenbeek 2007_ PTA | 260 | T | 100 | 13 | 201 | 11 | 18 | 6 |  | 3 | 8 | 1 |
| Ulrich 1989 | 261 |  | 83 | 13 | 52 |  | 5 | 1 |  |  |  | 0 |
| Ulrich 1989_PNoA | 261 |  | 77 | 17 | 48 |  | 2 | 1 |  |  |  | 0 |
| Unertl 1987 | 262 |  | 100 | 23 | 20 | 2 | 5 | 0 |  |  |  |  |
| Unertl 1987_PGA | 262 |  | 100 | 18 | 19 | 0 | 1 | 0 |  |  |  |  |
| van Delden 2012 | 263 |  | 100 | 11 | 45 | 6 |  |  |  |  |  |  |
| van Delden 2012_Azith | 263 |  | 100 | 10 | 47 | 2 |  |  |  |  |  |  |
| Verwaest 1997 | 264 |  | 100 | 19 | 185 | 7 |  | 0 |  | 1 |  | 4 |
| Verwaest 1997_ PTA | 264 |  | 100 | 22 | 200 | 10 |  | 0 |  | 6 |  | 0 |
| Verwaest 1997_OA | 264 |  | 100 | 17 | 193 | 2 |  | 1 |  | 2 |  | 0 |

**Table S4 (continued): Groups of antibiotic-based prophylaxis (=TAP±PPAP±antifungal) studies ^a^**

| Author & regimen | Ref | Notes | MV | LOS | Patients | VAP | | |  | Blood stream infection | | |
| --- | --- | --- | --- | --- | --- | --- | --- | --- | --- | --- | --- | --- |
|  |  |  | % | d | n | v_ps_n |  | v_can_n |  | b_ps_n |  | b_can_n |
| Wiener 1995 | 265 |  | 100 | 11 | 31 | 0 |  | 0 |  |  |  | 2 |
| Wiener 1995_ PGNy | 265 |  | 100 | 11 | 30 | 2 |  | 0 |  |  |  | 0 |
| Zobel 1991 | 266 | P | 100 | 13 | 25 | 2 |  |  |  | 0 |  | 1 |
| Zobel 1991_PGA | 266 | P | 100 | 14 | 25 | 0 |  |  |  | 0 |  | 0 |
|  |  |  |  |  |  |  |  |  |  |  |  |  |

Table S4: Footnotes

Notes; T = Data originating from a study for which the majority of ICU admission were for trauma; P = paediatric ICU; crf = group wide candidemia risk factor; e = number of episodes rather than patients

MV = percentage of group receiving mechanical ventilation; NS – Not stated; LOS is mean or median length of ICU stay; ICU-LOS is the ICU length of stay.

v_ps_n is the count of *Pseudomonas* VAP; v_can_n is the count of *Candida* isolates from patients with VAP.

b_ps_n is the count of *Pseudomonas* bacteremia; b_can_n is the count of Candidemia;

* De Smet 2009 [208] The *Pseudomonas* counts in this study are available only as a combined category count for gram negative non-fermentative GNB and are provided here for reference but are not used in the analysis. The Pseudomonas counts in the study of Oostdijk 2011 [222] come from the study by De Smet 2009 [208] and are taken as listed in Table 2 of Oostdijk 2011 [222] even though this Table appears to be mislabelled in comparison to the text and table 1 of Oostdijk 2011 [222] and in relation to the data in De Smet 2009 [208].

The control group in one study by Stoutenbeek [1987] appears also as the control group in another study by this Author [1984] and is used only once in the analysis here.

Several (n = 24) of these studies were cited in the following source systematic reviews.

- Minozzi S, Pifferi S, Brazzi L, Pecoraro V, Montrucchio G, D'Amico R. Topical antibiotic prophylaxis to reduce respiratory tract infections and mortality in adults receiving mechanical ventilation. Cochrane Database Syst Rev 2021, Issue 1. Art. No.: CD000022. DOI: 10.1002/14651858.CD000022.pub4.
- Pileggi C, Bianco A, Flotta D, Nobile CG, Pavia M. Prevention of ventilator-associated pneumonia, mortality and all intensive care unit acquired infections by topically applied antimicrobial or antiseptic agents: a meta-analysis of randomized controlled trials in intensive care units. *Crit Care* 2011; 15:R155.
- Silvestri L, Van Saene HK, Milanese M, Gregori D, Gullo A. Selective decontamination of the digestive tract reduces bacterial bloodstream infection and mortality in critically ill patients. Systematic review of randomized, controlled trials. J Hosp Infect. 2007;65(3):187-203.

TAP intervention regimens abbrevations; PTA (=P, topical polymyxin; T, topical tobramycin; A, topical amphotericin); PTA-Ctx (=P, topical polymyxin; T, topical tobramycin; A, topical amphotericin; Ctx, parenteral cephalosporin); P (P = polymyxin either aerosolized or topical); PNeV (P = polymyxin; Ne = Neomycin; V = Vancomycin); PGA-Ctx (=P, topical polymyxin; G, topical gentamicin; A, topical amphotericin; Ctx, parenteral cephalosporin); PTAM (=P, topical polymyxin; T, topical tobramycin; A, topical amphotericin; topical mupirocin); E (=E, topical erythromycin); PNoA-Ctx (=P, topical polymyxin; No, topical norfloxacin; A, topical amphotericin; Ctx, parenteral cephalsporin); PGV (=P, topical polymyxin; G, topical gentamicin; V, topical vancomycin); PGNy-Ctx (=P, topical polymyxin; G, topical gentamicin; Ny, topical nystatin; Ctx, parenteral cephalsporin); P-Ctx (=P, topical polymyxin; Ctx, parenteral cephalosporin); PTAV (=P, topical polymyxin; T, topical tobramycin; A, topical amphotericin; V, topical vancomycin); PGA (=P, topical polymyxin; G, topical gentamicin; A, topical amphotericin); PTNeA (=P, topical polymyxin; T, topical tobramycin; Ne, topical Neomycin; A, topical amphotericin); PGNy (=P, topical polymyxin; G, topical gentamicin; Ny, topical nystatin); PTA-Cz (=P, topical polymyxin; T, topical tobramycin; A, topical amphotericin; Cz, parenteral Ceftazidime).

**Table S5: Groups of studies of single drug antifungal (SAF) prophylaxis**

| Author & regimen | Ref | Notes | MV | LOS | Patients | RT Candida | | |  | Candidiemia | | |
| --- | --- | --- | --- | --- | --- | --- | --- | --- | --- | --- | --- | --- |
|  |  |  | % | d | n |  |  | v_can_n |  |  |  | b_can_n |
| Ables 2000 | 267 | crf | 95 | 8 | 59 |  |  |  |  |  |  | 0 |
| Ables 2000_Fluc | 267 | crf | 95 | 8 | 60 |  |  |  |  |  |  | 0 |
| Eggimann 1999 | 268 | crf |  |  | 20 |  |  |  |  |  |  | 2 |
| Eggimann 1999_Fluc | 268 | crf |  |  | 23 |  |  |  |  |  |  | 0 |
| Garbino 2002 | 269 |  | 100 | 8 | 101 |  |  | 0 |  |  |  | 9 |
| Garbino 2002_Fluc | 269 |  | 100 | 9 | 103 |  |  | 2 |  |  |  | 1 |
| Giglio 2012 | 270 |  | 100 | 15 | 50 |  |  | 0 |  |  |  | 0 |
| Giglio 2012_Ny | 270 |  | 100 | 15 | 49 |  |  | 0 |  |  |  | 0 |
| Jacobs 2003 | 271 |  |  | 11 | 39 |  |  |  |  |  |  | 1 |
| Jacobs 2003_Fluc | 271 |  |  | 11 | 32 |  |  |  |  |  |  | 0 |
| Lumbreras 1996_Ny | 272 | crf |  | 28 | 67 |  |  |  |  |  |  | 0 |
| Lumbreras 1996_Fluc | 272 | crf |  | 28 | 76 |  |  |  |  |  |  | 1 |
| Normand 2005 | 273 |  | 100 | 12 | 47 |  |  | 0 |  |  |  | 0 |
| Normand 2005_Ny | 273 |  | 100 | 12 | 51 |  |  | 0 |  |  |  | 0 |
| Ostrosky-Zeichner 2014 | 274 | crf | 100 | 7 | 102 |  |  |  |  |  |  | 7 |
| Ostrosky-Zeichner 2014_Casp | 274 | crf | 100 | 7 | 117 |  |  |  |  |  |  | 1 |
| Parizkova 2000 | 275 |  | 100 |  | 20 |  |  |  |  |  |  | 0 |
| Parizkova 2000_Fluc | 275 |  | 100 |  | 18 |  |  |  |  |  |  | 0 |
| Pelz_2001 | 276 | T | 87 | 5 | 130 |  |  |  |  |  |  | 3 |
| Pelz_2001_Fluc | 276 | T | 88 | 5 | 130 |  |  |  |  |  |  | 1 |
| Piarroux_2004 | 277 |  |  | 15 | 455 |  |  |  |  |  |  | 6 |
| Piarroux_2004_Fluc | 277 |  |  | 12 | 428 |  |  |  |  |  |  | 0 |
| Savino 1994 | 278 |  | 31 | 16 | 72 |  |  |  |  |  |  | 0 |
| Savino 1994_Clot | 278 |  | 23 | 12 | 80 |  |  |  |  |  |  | 0 |
| Savino 1994_Ket | 278 |  | 33 | 8 | 65 |  |  |  |  |  |  | 0 |
| Savino 1994_Ny | 278 |  | 39 | 15 | 75 |  |  |  |  |  |  | 0 |
| Savino 1994 | 278 | crf | 31 | 16 | 72 |  |  |  |  |  |  | 2 |
| Savino 1994_Clot | 278 | crf | 23 | 12 | 80 |  |  |  |  |  |  | 1 |
| Savino 1994_Ket | 278 | crf | 33 | 8 | 65 |  |  |  |  |  |  | 1 |
| Savino 1994_Ny | 278 | crf | 39 | 15 | 75 |  |  |  |  |  |  | 5 |
| Schuster 2008 | 279 | crf |  | 12 | 127 |  |  |  |  |  |  | 2 |
| Schuster 2008_Fluc | 279 | crf |  | 12 | 122 |  |  |  |  |  |  | 0 |
|  |  |  |  |  |  |  |  |  |  |  |  |  |

Table S5: Footnotes

T = Data originating from a trauma ICU; crf = group wide candidemia risk factor; MV = percentage of group receiving mechanical ventilation; NS – Not stated; LOS is mean or median length of ICU stay; ICU-LOS is the ICU length of stay.

RT Candida is respiratory tract candida (v_can_n; the count of *Candida* isolates from patients with VAP); b_can_n is the count of Candidemia

Several (n = 24) of these studies were cited in the following source systematic reviews [288-291].

**Table 6: Review of effect sizes in the literature** ^a, b^

|  | Effect size | 95% CI | notes | Ref |
| --- | --- | --- | --- | --- |
| Anti-septics |  |  |  |  |
| Overall VAP |  |  |  |  |
|  | RR: 0.78 | 0.60 – 1.02 (13) | Excl cardiac surgery | 281 |
|  | RR: 0.67 | 0.47 – 0.97 (13) | Chlorhexidine | 282 |
|  | RR: 0.66 | 0.51 – 0.85 (22) | Nosocomial pneumonia | 283 |
|  |  |  |  |  |
| Overall bactremia | |  |  |  |
|  | OR: 0.74 | 0.37 – 1.50 (5) | Chlorhexidine | 284 |
|  |  |  |  |  |
| Antibiotic based | |  |  |  |
| Overall VAP |  |  |  |  |
|  | OR: 0.35 | 0.30 – 0.42 (26) |  | 285 |
|  | RR: 0.43 | 0.35 – 0.53 (18) | TAP + PPAP | 286 |
|  | RR: 0.57 | 0.44 – 0.74 (19) | TAP alone (incl duplex) | 286 |
|  | RR: 0.59 | 0.36 – 0.69 (13) | TAP alone (excl duplex) | 286 |
|  |  |  |  |  |
| Bacteraemia | |  |  |  |
|  | OR: 0.48 | 0.37 – 0.64 (16) | Overall bacteraemia | 285 |
|  | OR: 0.30 | 0.16 – 0.56 (8) | Gram Neg (TAP + PPAP) | 287 |
|  | OR: 1.04 | 0.69 – 1.57 (8) | Gram Pos (TAP + PPAP) | 287 |
|  | OR: 0.63 | 0.46 – 0.87 (16) | Overall bacteremia | 287 |
|  |  |  |  |  |
| Candida infections | |  |  |  |
|  | OR: 0.39 | 0.21 – 0.72 (11) | Candidemia | 288 |
|  | OR: 0.29 | 0.18 – 0.45 (15) | Invasive fungal infections | 288 |
|  | OR: 0.32 | 0.18 – 0.57 (5) | (Overall fungal infections) | 289 |
|  |  |  |  |  |
| Single anti-fungal (SAF) | |  |  |  |
| Candidemia |  |  |  |  |
|  | OR: 0.32 | 0.12 – 0.82 (6) |  | 288 |
|  |  |  |  |  |
| Other candida infections | |  |  |  |
|  | OR: 0.54 | 0.39 – 0.75 (10) | Invasive fungal infections | 288 |
|  | OR: 0.69 | 0.37 – 1.79 (5) | superficial fungal infections | 290 |
|  | OR: 0.57 | 0.39 – 0.83 (18) | Invasive fungal infections | 290 |
|  | OR: 0.71 | 0.52 – 0.97 (12) | fungal colonization | 290 |
|  | RR: 0.46 | 0.31 – 0.68 (9) | Invasive fungal infections | 291 |
|  |  |  |  |  |

References

1. A'Court CH, Garrard CS, Crook D, Bowler I, Conlon C, Peto T, Anderson E: Microbiological lung surveillance in mechanically ventilated patients, using non-directed bronchial lavage and quantitative culture. Q J Med. 1993;86:635-48.
2. Alvarez-Lerma F, ICU-acquired Pneumonia Study Group. Modification of empiric antibiotic treatment in patients with pneumonia acquired in the intensive care unit. *Intens Care Med.* 1996;22(5):387-94.
3. Antonelli M, Moro ML, Capelli O, De Blasi RA, D’Errico RR, Conti G, Bufi M, Gasparetto A: Risk factors for early onset pneumonia in trauma patients. Chest. 1994;105:224-228
4. Apostolopoulou E, Bakakos P, Katostaras T, Gregorakos L: Incidence and risk factors for ventilator-associated pneumonia in 4 multidisciplinary intensive care units in Athens, Greece. Respir Care. 2003;48: 681-688.
5. Arumugam SK, Mudali I, Strandvik G, El-Menyar A, Al-Hassani A, Al-Thani H. Risk factors for ventilator-associated pneumonia in trauma patients: A descriptive analysis. World J Emerg Med. 2018;9(3):203.
6. Azoulay E, Timsit JF, Tafflet M, de Lassence A, Darmon M, Zahar JR, Schlemmer B. *Candida* colonization of the respiratory tract and subsequent pseudomonas ventilator-associated pneumonia. *Chest* 2006;129(1):110-117.
7. Bailly S, Bouadma L, Azoulay E, Orgeas MG, Adrie C, Souweine B, Schwebel C, Maubon D, Hamidfar-Roy R, Darmon M, Wolff M. Failure of empirical systemic antifungal therapy in mechanically ventilated critically ill patients. *Am J Respir Crit Care Med.* 2015;191(10):1139-46.
8. Bekaert M, Timsit JF, Vansteelandt S, Depuydt P, Vésin A, Garrouste-Orgeas M, Decruyenaere J, Clec'h C, Azoulay E, Benoit D. Attributable mortality of ventilator-associated pneumonia: a reappraisal using causal analysis. *Am J Respir Crit Care Med*. 2011;184(10):1133-9.
9. Bercault N, Boulain T: Mortality rate attributable to ventilator-associated nosocomial pneumonia in an adult intensive care unit: a prospective case-control study. Crit Care Med. 2001;29:2303-2309
10. Bercault N, Wolf M, Runge I, et al. Intrahospital transport of critically ill ventilated patients: a risk factor for ventilator-associated pneumonia--a matched cohort study. *Crit Care Med* 2005;33:2471-8.
11. Berrouane Y, Daudenthun I, Riegel B, Emery MN, Martin G, Krivosic R, Grandbastien B. Early onset pneumonia in neurosurgical intensive care unit patients. J Hosp Infect. 1998;40(4):275-80.
12. Blot S, Koulenti D, Dimopoulos G, Martin C, Komnos A, Krueger WA, Spina G, Armaganidis A, Rello J. Prevalence, risk factors, and mortality for ventilator-associated pneumonia in middle-aged, old, and very old critically ill patients. Crit Care Med. 2014;42(3):601-9.
13. Bochicchio GV, Joshi M, Bochicchio K, Tracy K, Scalea TM: A time-dependent analysis of intensive care unit pneumonia in trauma patients. J Trauma. 2004;56:296-301.
14. Bonten MJ, Gaillard CA, van Tiel FH, Smeets HG, van der Geest S, Stobberingh EE: The stomach is not a source for colonization of the upper respiratory tract and pneumonia in ICU patients. Chest. 1994;105(3):878-84.
15. Boots RJ, Phillips GE, George N, Faoagali JL. Surveillance culture utility and safety using low‐volume blind bronchoalveolar lavage in the diagnosis of ventilator‐associated pneumonia. Respirology. 2008;13:87-96.
16. Bornstain C, Azoulay E, De Lassence A, Cohen Y, Costa MA, Mourvillier B, Descorps-Declere A, Garrouste-Orgeas M, Thuong M, Schlemmer B, Timsit JF: Sedation, sucralfate, and antibiotic use are potential means for protection against early-onset ventilator-associated pneumonia. Clin Infect Dis. 2004;38(10):1401-8.
17. Borzotta AP, Beardsley K (1999) Candida infections in critically ill trauma patients: a retrospective case-control study. Arch Surg 134(6):657-665
18. Braun SR, Levin AB, Clark KL. Role of corticosteroids in the development of pneumonia in mechanically ventilated head-trauma victims. Crit Care Med 1986;14:198-201
19. Bregeon F, Papazian L, Visconti A, Gregoire R, Thirion X, Gouin F: Relationship of microbiologic diagnostic criteria to morbidity and mortality in patients with ventilator-associated pneumonia. JAMA. 1997;277: 655-662
20. Bronchard R, Albaladejo P, Brezac G, et al. Early onset pneumonia: risk factors and consequences in head trauma patients. *Anesthesiology* 2004;100:234-9.
21. Cade JF, McOwat E, Siganporia R, Keighley C, Presneill J, Sinickas V: Uncertain relevance of gastric colonization in the seriously ill. Intensive Care Med. 1992;18:210-217
22. Cavalcanti M, Ferrer M, Ferrer R, Morforte R, Garnacho A, Torres A: Risk and prognostic factors of ventilator-associated pneumonia in trauma patients. Crit Care Med. 2006;34:1067-1072
23. Cendrero JA, Solé-Violán J, Benitez AB, Catalán JN, Fernández JA, Santana PS, de Castro FR: Role of different routes of tracheal colonization in the development of pneumonia in patients receiving mechanical ventilation. Chest. 1999;116:462-470
24. Chaari A, El Habib M, Ghdhoun H, Algia NB, Chtara K, Hamida CB, Chelly H, Bahloul M, Bouaziz M. Does low-dose hydrocortisone therapy prevent ventilator-associated pneumonia in trauma patients? Am J Therap. 2015;22(1):22-8.
25. Charles PE, Dalle F, Aube H, Doise JM, Quenot JP, Aho LS, Blettery B (2005) Candida spp. colonization significance in critically ill medical patients: a prospective study. Intensive Care Med 31(3):393-400.
26. Chastre J, Trouillet JL, Vuagnat A, Joly-Guillou ML, Clavier H, Dombret MC, Gibert C: Nosocomial pneumonia in patients with acute respiratory distress syndrome. *Am J Respir Crit Care Med.* 1998;157:1165-1172
27. Chevret S, Hemmer M, Carlet J: Incidence and risk factors of pneumonia acquired in intensive care units. Results from a multicenter prospective study on 996 patients. European Cooperative Group on Nosocomial Pneumonia. Intensive Care Med. 1993;19:256-264
28. Combes P, Fauvage B, Oleyer C. Nosocomial pneumonia in mechanically ventilated patients, a prospective randomised evaluation of the Stericath closed suctioning system. *Intensive Care Med* 2000;26:878-82.
29. Cook A, Norwood S, Berne J: Ventilator-associated pneumonia is more common and of less consequence in trauma patients compared with other critically ill patients. J Trauma Acute Care Surg. 2010;69(5):1083-91.
30. Craven DE, Kunches LM, Lichtenberg DA, Kollisch NR, Barry MA, Heeren TC, McCabe WR: Nosocomial infection and fatality in medical and surgical intensive care unit patients. Arch Intern Med. 1988;148:1161-1168
31. Daschner F, Kappstein I, Schuster F, Scholz R, Bauer E, Jooβens D, Just H: Influence of disposable ('Conchapak') and reusable humidifying systems on the incidence of ventilation pneumonia. J Hosp Infect. 1988;11:161-168
32. De Waele JJ, Vogelaers D, Blot S Colardyn F (2003) Fungal infections in patients with severe acute pancreatitis and the use of prophylactic therapy. Crit Care 2003: 7(2) 1.
33. De Latorre FJ, Pont T, Ferrer A, Rosselló J, Palomar M, Planas M: Pattern of tracheal colonization during mechanical ventilation. Am J Respir Crit Care Med. 1995;152:1028-1033
34. De Santis V, Gresoiu M, Corona A, Wilson AP, Singer M. Bacteraemia incidence, causative organisms and resistance patterns, antibiotic strategies and outcomes in a single university hospital ICU: continuing improvement between 2000 and 2013. J Antimicrob Chemoth. 2015;70(1):273-8.
35. El-Masri MM, Hammad TA, McLeskey SW, Joshi M, Korniewicz DM. Predictors of nosocomial bloodstream infections among critically ill adult trauma patients. *Infect Cont & Hosp Epidemiol.* 2004;25(8):656-63.
36. Ensminger SA, Wright RS, Baddour LM, Afess B: Suspected ventilator-associated pneumonia in cardiac patients admitted to the coronary care unit. Mayo Clin Proc. 2006;81:32–35
37. Ertugrul BM, Yildirim A, Ay P, Oncu S, Cagatay A, Cakar N, Ertekin C, Ozsut H, Eraksoy H, Calangu S. Ventilator-associated pneumonia in surgical emergency intensive care unit. Saudi Med J. 2006;27(1):52.
38. Esteve F, Pujol M, Limon E, Saballs M, Argerich MJ, Verdaguer R, Manez R, Ariza X, Gudiol F. Bloodstream infection related to catheter connections: a prospective trial of two connection systems. *J Hosp Infect*. 2007;67(1):30-4.
39. Evans HL, Zonies DH, Warner KJ, Bulger EM, Sharar SR, Maier RV, Cuschieri J. Timing of intubation and ventilator-associated pneumonia following injury. Arch Surg. 2010;145(11):1041-6.
40. Ewig S, Torres A, El-Ebiary M, Fàbregas N, Hernandez C, Gonzalez J, Nicolas JM, Soto L: Bacterial colonization patterns in mechanically ventilated patients with traumatic and medical head injury. Incidence, risk factors, and association with ventilator-associated pneumonia. Am J Respir Crit Care Med. 1999;159:188-198
41. Fabian TC, Boucher BA, Croce MA, Kuhl DA, Janning SW, Coffey BC, Kudsk KA: Pneumonia and stress ulceration in severely injured patients: a prospective evaluation of the effects of stress ulcer prophylaxis. Arch Surg. 1993;128(2):185-92.
42. Fagon JY, Chastre J, Domart Y, Trouillet JL, Pierre J, Darne C, Gibert C: Nosocomial pneumonia in patients receiving continuous mechanical ventilation. Prospective analysis of 52 episodes with use of a protected specimen brush and quantitative culture techniques. *Am Rev Respir Dis* 1989;139:877-884.
43. Ferreira D, Grenouillet F, Blasco G, Samain E, Hénon T, Dussaucy A, Millon L, Mercier M, Pili-Floury S. Outcomes associated with routine systemic antifungal therapy in critically ill patients with Candida colonization. Intens care Med. 2015;41(6):1077-88.
44. Gacouin A, Barbarot N, Camus C, Salomon S, Isslame S, Marque S, Lavoué S, Donnio PY, Thomas R, Le Tulzo Y. Late-onset ventilator-associated pneumonia in nontrauma intensive care unit patients. Anesth Analg. 2009;109(5):1584-90.
45. García-Garmendia JL, Ortiz-Leyba C, Garnacho-Montero J, Jiménez-Jiménez FJ, Pérez-Paredes C, Barrero-Almodóvar AE, Miner MG. Risk factors for Acinetobacter baumannii nosocomial bacteremia in critically ill patients: a cohort study. *Clin Infect Dis*. 2001;33(7):939-46.
46. Garrouste-Orgeas M, Chevret S, Arlet G, Marie O, Rouveau M, Popoff N, Schlemmer B: Oropharyngeal or gastric colonization and nosocomial pneumonia in adult intensive care unit patients. A prospective study based on genomic DNA analysis. Am J Respir Crit Care Med. 1997;156(5):1647-56.
47. Garrouste-Orgeas M, Timsit JF, Tafflet M, Misset B, Zahar JR, Soufir L, Carlet J: Excess risk of death from intensive care unit—acquired nosocomial bloodstream infections: a reappraisal. *Clin Infect Dis* 2006, 42:1118-1126.
48. George DL, Falk PS, Wunderink RG, Leeper Jr KV, Meduri GU, Steere EL, Glen Mayhall C: Epidemiology of ventilator-acquired pneumonia based on protected bronchoscopic sampling. Am J Respir Crit Care Med. 1998;158:1839-1847
49. Georges H, Leroy O, Guery B, Alfandari S, Beaucaire G: Predisposing factors for nosocomial pneumonia in patients receiving mechanical ventilation and requiring tracheotomy. Chest. 2000;118:767–774.
50. Giamarellos-Bourboulis EJ, Bengmark S, Kannellakopoulou K, Kotzampassi K: Pro- and synbiotics to control inflammation and infection in patients with multiple injuries. *J Trauma* 2009;67:815-821.
51. Giard M, Lepape A, Allaouchiche B, Guerin C, Lehot JJ, Robert MO, Vanhems P: Early-and late-onset ventilator-associated pneumonia acquired in the intensive care unit: comparison of risk factors. *J Crit Care* 2008, 23:27-33.
52. Gruson D, Hilbert G, Vargas F, Valentino R, Bebear C, Allery A, Bebear C, Gbikpi-Benissan GE, Cardinaud JP: Rotation and restricted use of antibiotics in a medical intensive care unit: impact on the incidence of ventilator-associated pneumonia caused by antibiotic-resistant gram-negative bacteria. Am J Respir Crit Care Med. 2000, 162(3):837-43.
53. Gruson D, Hilbert G, Vargas F, Valentino R, Bui N, Pereyre S, Bebear C, Bebear CM, Gbikpi-Benissan G: Strategy of antibiotic rotation: long-term effect on incidence and susceptibilities of Gram-negative bacilli responsible for ventilator-associated pneumonia. Crit Care Med. 2003;31:1908-1914.
54. Guérin C, Girard R, Chemorin C, De Varax R, Fournier G: Facial mask noninvasive mechanical ventilation reduces the incidence of nosocomial pneumonia. Intens care Med. 1997;23(10):1024-32.
55. Gursel G, Aydogdu M, Nadir Ozis T, Tasyurek S. Comparison of the value of initial and serial endotracheal aspirate surveillance cultures in predicting the causative pathogen of ventilator-associated pneumonia. Scand J Infect Dis. 2010, 42:341-346.
56. Heyland DK, Cook DJ, Schoenfeld PS, Frietag A, Varon J, Wood G: The effect of acidified enteral feeds on gastric colonization in critically ill patients: results of a multicenter randomized trial. Canadian Critical Care Trials Group. Crit Care Med. 1999;27:2399-2406
57. Holzapfel L, Chevret S, Madinier G, Ohen F, Demingeon G, Coupry A, Chaudet M: Influence of long-term oro- or nasotracheal intubation on nosocomial maxillary sinusitis and pneumonia: results of a prospective, randomized, clinical trial. Crit Care Med. 1993;21:1132-1138
58. Huang SS, Septimus E, Kleinman K, Moody J, Hickok J, Avery TR, Lankiewicz J, Gombosev A, Terpstra L, Hartford F, Hayden MK. Targeted versus universal decolonization to prevent ICU infection. *N Engl J Med*. 2013;368(24):2255-65.
59. Hugonnet S, Uçkay I, Pittet D Staffing level: a determinant of late-onset ventilator-associated pneumonia. Crit Care. 2007;11(4):R80
60. Hyllienmark P, Gardlund B, Persson JO, Ekdahl K. Nosocomial pneumonia in the ICU: a prospective cohort study. Scand J Infect Dis. 2007;39:676-82.
61. Hyllienmark P, Brattström O, Larsson E, Martling CR, Petersson J, Oldner A: High incidence of post‐injury pneumonia in intensive care‐treated trauma patients. Acta Anaesthesiologica Scandinavica. 2013;57(7):848-54.
62. Ibáñez J, Peñafiel A, Marsé P, Jordá R, Raurich JM, Mata F: Incidence of gastroesophageal reflux and aspiration in mechanically ventilated patients using small-bore nasogastric tubes. J Parenteral and Enteral Nutrition. 2000;24(2):103-6.
63. Ibrahim EH, Ward S, Sherman G, Kollef MH: A comparative analysis of patients with early-onset vs late-onset nosocomial pneumonia in the ICU setting. Chest. 2000;117:1434-1442
64. Ibrahim EH, Sherman G, Ward S, Fraser VJ, Kollef MH. The influence of inadequate antimicrobial treatment of bloodstream infections on patient outcomes in the ICU setting. Chest. 2000;118(1):146-55.
65. Ibrahim EH, Tracy L, Hill C, et al. The occurrence of ventilator-associated pneumonia in a community hospital: risk factors and clinical outcomes. *Chest* 2001;120:555-61.
66. Jacobs S, Chang RW, Lee B, Bartlett FW: Continuous enteral feeding: a major cause of pneumonia among ventilated intensive care unit patients*. JPEN J Parenter Enteral Nutr* 1990;14:353-6.
67. Jaillette E, Nseir S: Relationship between inhaled β2-agonists and ventilator-associated pneumonia: A cohort study. Critical Care Med. 2011;39(4):725-30.
68. Jensen JUS, Hein L, Lundgren B, Bestle MH, Mohr T, Andersen MH, Procalcitonin And Survival Study Group (2015) Invasive *Candida* Infections and the Harm From Antibacterial Drugs in Critically Ill Patients: Data From a Randomized, Controlled Trial to Determine the Role of Ciprofloxacin, Piperacillin-Tazobactam, Meropenem, and Cefuroxime. *Crit Care Medicine* 2015;43(3):594-602.
69. Jiménez P, Torres A, Rodríguez-Roisin R, de la Bellacasa JP, Aznar R, Gatell JM, Agustí-Vidal A: Incidence and etiology of pneumonia acquired during mechanical ventilation. Crit Care Med. 1989;17:882-5.
70. Kautzky S, Staudinger T, Presterl E. Invasive Candida infections in patients of a medical intensive care unit. Wiener klinische Wochenschrift. 2015;127(3-4):132-42.
71. Ko HK, Yu WK, Lien TC, Wang JH, Slutsky AS, Zhang H, Kou YR. Intensive care unit-acquired bacteremia in mechanically ventilated patients: clinical features and outcomes. *PloS one*. 2013;8(12):e83298.
72. Kollef MH: Ventilator-associated pneumonia. A multivariate analysis. JAMA. 1993;270:1965-70.
73. Kollef MH, Silver P, Murphy DM, Trovillion E: The effect of late-onset ventilator-associated pneumonia in determining patient mortality. Chest. 1995;108: 1655-62.
74. Kollef MH, Shapiro SD, Fraser VJ, Silver P, Murphy DM, Trovillion E, Hearns ML, Richards RD, Cracchilo L, Hossin L: Mechanical ventilation with or without 7-day circuit changes. A randomized controlled trial. Ann Intern Med.1995;123:168–174
75. Kollef MH, Von Harz B, Prentice D, Shapiro SD, Silver P, John RS, Trovillion E: Patient transport from intensive care increases the risk of developing ventilator-associated pneumonia. Chest. 1997;112(3):765-773.
76. Kollef MH, Vlasnik JO, Sharpless L, Pasque C, Murphy D, Fraser V. Scheduled change of antibiotic classes: a strategy to decrease the incidence of ventilator-associated pneumonia. Am J Resp Crit Care Med. 1997;156(4):1040-8.
77. Kollef MH, Chastre J, Fagon JY, François B, Niederman MS, Rello J, Torres A, Vincent JL, Wunderink RG, Go KW, Rehm C. Global prospective epidemiologic and surveillance study of ventilator-associated pneumonia due to Pseudomonas aeruginosa. Crit care med. 2014;42(10):2178-87.
78. Koss WG, Khalili TM, Lemus JF, Chelly MM, Margulies DR, Shabot MM: Nosocomial pneumonia is not prevented by protective contact isolation in the surgical intensive care unit. Am Surg. 2001;67:1140-4.
79. Kunac A, Sifri ZC, Mohr AM, Horng H, Lavery RF, Livingston DH: Bacteremia and Ventilator-Associated Pneumonia: A Marker for Contemporaneous Extra-Pulmonic Infection. Surg Infect. 2014;15:77-83.
80. Lambert ML, Suetens C, Savey A, Palomar M, Hiesmayr M, Morales I, Agodi A, Frank U, Mertens K, Schumacher M, Wolkewitz M. Clinical outcomes of health-care-associated infections and antimicrobial resistance in patients admitted to European intensive-care units: a cohort study. Lancet Infect Dis. 2011;11(1):30-8.
81. Laupland KB, Zygun DA, Davies HD, Church DL, Louie TJ, Doig CJ Population-based assessment of intensive care unit-acquired bloodstream infections in adults: incidence, risk factors, and associated mortality rate. *Crit Care Med* 2002;30:2462-2467.
82. Laupland KB, Kirkpatrick AW, Church DL, Ross T, Gregson DB Intensive-care-unit-acquired bloodstream infections in a regional critically ill population. *J Hosp Infect* 2004;58(2): 137-145.
83. León C, Ruiz-Santana S, Saavedra P, Almirante B, Nolla-Salas J, Álvarez-Lerma F, Garnacho-Montero J, León MÁ. A bedside scoring system (“Candida score”) for early antifungal treatment in nonneutropenic critically ill patients with Candida colonization. Crit Care Med. 2006 Mar 1;34(3):730-7.
84. León C, Ruiz-Santana S, Saavedra P, Galván B, Blanco A, Castro C, Balasini C, Utande-Vázquez A, de Molina FJ, Blasco-Navalproto MA, López MJ. Usefulness of the “Candida score” for discriminating between Candida colonization and invasive candidiasis in non-neutropenic critically ill patients: a prospective multicenter study. Crit Care Med. 2009;37(5):1624-33.
85. León C, Ruiz-Santana S, Saavedra P, Castro C, Loza A, Zakariya I, Úbeda A, Parra M, Macías D, Tomás JI, Rezusta A. Contribution of Candida biomarkers and DNA detection for the diagnosis of invasive candidiasis in ICU patients with severe abdominal conditions. Crit Care. 2016;20(1):149.
86. Lepelletier D, Roquilly A, Mahe PJ, Loutrel O, Champin P, Corvec S, Naux E, Pinaud M, Lejus C, Asehnoune K. Retrospective analysis of the risk factors and pathogens associated with early-onset ventilator-associated pneumonia in surgical-ICU head-trauma patients. J Neurosurg Anesthesiol. 2010;22(1):32-7.
87. Li Z, Jiang C, Dong D, Zhang L, Tian Y, Ni Q, Mao E, Peng Y. The correlation between Candida colonization of distinct body sites and invasive candidiasis in emergency intensive care units: statistical and molecular biological analysis. Mycopathologia. 2016;181(7-8):475-84.
88. Luna CM, Blanzaco D, Niederman MS, et al. Resolution of ventilator-associated pneumonia: prospective evaluation of the clinical pulmonary infection score as an early clinical predictor of outcome. Crit Care Med. 2003;31:676-682.
89. Luyt CE, Guérin V, Combes A, Trouillet JL, Ayed SB, Bernard M, Gibert C, Chastre J: Procalcitonin kinetics as a prognostic marker of ventilator-associated pneumonia. Am J Respir Crit Care Med. 2005;171:48-53.
90. Magnason S, Kristinsson KG, Stefansson T, Erlendsdottir H, Jonsdottir K, Kristjansson M, Gudmundsson S: Risk factors and outcome in ICU‐acquired infections. Acta Anaesthesiol Scand. 2008;52:1238-1245
91. Mahul P, Auboyer C, Jospe R, Ros A, Guerin C, el Khouri Z, Galliez M, Dumont A, Gaudin O: Prevention of nosocomial pneumonia in intubated patients respective role of mechanical subglottic secretions drainage and stress ulcer prophylaxis. Intensive Care Med. 1992;18:20-25
92. Makris D, Manoulakas E, Komnos A, Papakrivou E, Tzovaras N, Hovas A, Zintzaras E, Zakynthinos E. Effect of pravastatin on the frequency of ventilator-associated pneumonia and on intensive care unit mortality: open-label, randomized study. Crit care med. 2011;39(11):2440-6.
93. Markowicz P, Wolff M, Djedaini K, Cohen Y, Chastre J, Delclaux C: Multicenter prospective study of ventilator-associated pneumonia during acute respiratory distress syndrome. Incidence, prognosis, and risk factors. ARDS Study Group. Am J Respir Crit Care Med. 2000;161:1942-8.
94. Memish ZA, Cunningham G, Oni GA, et al. The incidence and risk factors of ventilator-associated pneumonia in a Riyadh hospital. Infect Control Hosp Epidemiol. 2000;21:271-3.
95. Michel F, Franceschini B, Berger P, Arnal JM, Gainnier M, Sainty JM, Papazian L. Early antibiotic treatment for BAL-confirmed ventilator-associated pneumonia: a role for routine endotracheal aspirate cultures. Chest. 2005;127(2):589-97.
96. Mitsogianni M, Vasileiadis I, Parisi M, Tzanis G, Kampisiouli E, Psaroudaki Z, Perivolioti E, Fountoulis K, Routsi C, Nanas S, Tsiodras S. A Multifaceted Intervention Program to Prevent Bloodstream Infection in an IntensiveCare Unit. *Health Science J*. 2016;10(2):1.
97. Moine P, Timsit JF, De Lassence A, Troché G, Fosse JP, Alberti C, Cohen Y: Mortality associated with late-onset pneumonia in the intensive care unit: results of a multi-center cohort study. Intensive Care Med. 2002;28:154-163
98. Montecalvo MA, Steger KA, Farber HW: Nutritional outcome and pneumonia in critical care patients randomized to gastric versus jejunal tube feedings. The Critical Care Research Team. *Crit Care Med* 1992, 20:1377-1387.
99. Myny D, Depuydt P, Colardyn F, Blot S: Ventilator-associated pneumonia in a tertiary care ICU analysis of risk factors for acquisition and mortality. Acta Clin Belg. 2005;60:114-121.
100. Nguile-Makao M, Zahar JR, Français A, Tabah A, Garrouste-Orgeas M, Allaouchiche B, Goldgran-Toledano D, Azoulay E, Adrie C, Jamali S, Clec’h C. Attributable mortality of ventilator-associated pneumonia: respective impact of main characteristics at ICU admission and VAP onset using conditional logistic regression and multi-state models. Intens care med. 2010;36(5):781-9.
101. Nielsen SL, Røder B, Magnussen P, Engquist A, Frimodt-møller N. Nosocomial pneumonia in an intensive care unit in a Danish university hospital: incidence, mortality and etiology. Scand J Infect Dis. 1992;24:65-70.
102. Nseir S, Di Pompeo C, Soubrier S, Cavestri B, Jozefowicz E, Saulnier F, Durocher A: Impact of ventilator-associated pneumonia on outcome in patients with COPD. Chest. 2005;128(3):1650-1656.
103. Nseir S, Jozefowicz E, Cavestri B, Sendid B, Di Pompeo C, Dewavrin F, Durocher A. Impact of antifungal treatment on Candida–Pseudomonas interaction: a preliminary retrospective case–control study. *Intensive Care Med* 2007;33(1):137-142.
104. Orsi GB, Giuliano S, Franchi C, Ciorba V, Protano C, Giordano A, Rocco M, Venditti M. Changed epidemiology of ICU acquired bloodstream infections over 12 years in an Italian teaching hospital. *Minerva Anestesiol.* 2015;81(9):980-8.
105. Osmon S, Warren D, Seiler SM, Shannon W, Fraser VJ, Kollef MH: The influence of infection on hospital mortality for patients requiring >48 h of intensive care. *Chest* 2003, 124:1021-1029.
106. [OUTCOMEREA] Saied WI, Mourvillier B, Cohen Y, Ruckly S, Reignier J, Marcotte G, Siami S, Bouadma L, et al on behalf of the OUTCOMEREA study group. A comparison of the mortality risk associated with ventilator-acquired bacterial pneumonia and nonventilator ICU-acquired bacterial pneumonia. Crit care med. 2019;47:345-52.
107. Papazian L, Bregeon F, Thirion X, Gregoire R, Saux P, Denis JP, Perin G, Charrel J, Dumon JF, Affray JP, Gouin F: Effect of ventilator-associated pneumonia on mortality and morbidity. Am J Respir Crit Care Med. 1996;154:91-7.
108. Petri MG, König J, Moecke HP, Gramm HJ, Barkow H, Kujath P, Dennhart R, Lode H. Epidemiology of invasive mycosis in ICU patients: a prospective multicenter study in 435 non-neutropenic patients. *Intensive Care Med* 1997;23(3):317-325.
109. Potgieter PD, Linton DM, Oliver S, Forder AA: Nosocomial infections in a respiratory intensive care unit. Crit Care Med. 1987;15:495-498
110. Prowle JR, Echeverri JE, Ligabo EV, Sherry N, Taori GC, Crozier TM, Bellomo R. Acquired bloodstream infection in the intensive care unit: incidence and attributable mortality. Crit Care 2011;15(2):R100.
111. Rello J, Quintana E, Ausina V, Castella J, Luquin M, Net A, Prats G: Incidence, etiology, and outcome of nosocomial pneumonia in mechanically ventilated patients. Chest. 1991;100:439-444
112. Rello J, Ausina V, Castella J, et al Nosocomial respiratory tract infections in multiple trauma patients. Influence of level of consciousness with implications for therapy. Chest 1992;102:525-529
113. Rello J, Ricart M, Mirelis B, Quintana E, Gurgui M, Net A, Prats, G: Nosocomial bacteremia in a medical-surgical intensive care unit: epidemiologic characteristics and factors influencing mortality in 111 episodes. *Intensive Care Med* 1994;20:94-98.
114. Rello J, Ausina V, Ricart M, Puzo C, Quintana E, Net A, Prats G. Risk factors for infection by *Pseudomonas aeruginosa* in patients with ventilator-associated pneumonia. *Intens Care Med*. 1994;20(3):193-8.
115. Rello J, Ollendorf DA, Oster G, et al. Epidemiology and outcomes of ventilator-associated pneumonia in a large US database. Chest 2002;122:2115-2121
116. Rello J, Lorente C, Diaz E, et al. Incidence, etiology, and outcome of nosocomial pneumonia in ICU patients requiring percutaneous tracheotomy for mechanical ventilation. *Chest*. 2003;124:2239-2243.
117. Resende MM, Monteiro SG, Callegari B, Figueiredo PM, Monteiro CR, Monteiro-Neto V. Epidemiology and outcomes of ventilator-associated pneumonia in northern Brazil: an analytical descriptive prospective cohort study. BMC infect Dis 2013;13(1): 119
118. Reusser P, Zimmerli W, Scheidegger D, Marbet GA, Buser M, Gyr K: Role of gastric colonization in nosocomial infections and endotoxemia: a prospective study in neurosurgical patients on mechanical ventilation. J Infect Dis. 1989;160:414-421
119. Rincón-Ferrari MD, Flores-Cordero JM, Leal-Noval SR, Murillo-Cabezas F, Cayuelas A, Muñoz-Sánchez MA, Sánchez-Olmedo JI: Impact of ventilator-associated pneumonia in patients with severe head injury. J Trauma Acute Care Surg. 2004;57(6):1234-40.
120. Rodrigues PM, Neto C, Santos LR, Knibel MF. Ventilator-associated pneumonia: epidemiology and impact on the clinical evolution of ICU patients. Jornal brasileiro de pneumologia. 2009 Nov;35(11):1084-91.
121. Rodriguez JL, Gibbons KJ, Bitzer LG, Dechert RE, Steinberg SM, Flint LM: Pneumonia: incidence, risk factors, and outcome in injured patients. J Trauma. 1991;31: 907-12.
122. Ruiz-Santana S, Garcia Jimenez A, Esteban A, et al. ICU pneumonias: a multi-institutional study. *Crit Care Med*. 1987;15:930-932.
123. Salata RA, Lederman MM, Shlaes DM, Jacobs MR, Eckstein E, Tweardy D, Toossi Z, Chmielewski R, Marino J, King CH: Diagnosis of nosocomial pneumonia in intubated, intensive care unit patients. Am Rev Respir Dis. 1987;135:426-432
124. Shahin J, Bielinski M, Guichon C, Flemming C, Kristof AS Suspected ventilator-associated respiratory infection in severely ill patients: a prospective observational study. Crit Care 2013;17(5): R251
125. Sofianou DC, Constandinidis TC, Yannacou M, Anastasiou H, Sofianos E: Analysis of risk factors for ventilator-associated pneumonia in a multidisciplinary intensive care unit. *Eur J Clin Microbiol Infect Dis* 2000, 19:460-463.
126. Stéphan F, Mabrouk N, Decailliot F, Delclaux C, Legrand P: Ventilator-associated pneumonia leading to acute lung injury after trauma: importance of *Haemophilus influenzae*. Anesthesiology. 2006;104: 235-41.
127. Tan X, Zhu S, Yan D, Chen W, Chen R, Zou J, Yan J, Zhang X, Farmakiotis D, Mylonakis E. Candida spp. airway colonization: A potential risk factor for Acinetobacter baumannii ventilator-associated pneumonia. Med Mycol. 2016:myw009.
128. Tejada Artigas AT, Dronda SB, Vallés EC, Marco JM, Usón MC, Figueras P, Suarez FJ, Hernandez A: Risk factors for nosocomial pneumonia in critically ill trauma patients. Crit Care Med. 2001;29:304-9.
129. Thompson DS. Estimates of the rate of acquisition of bacteraemia and associated excess mortality in a general intensive care unit: a 10 year study. *J Hosp Infect*. 2008;69(1):56-61.
130. Timsit JF, Chevret S, Valcke J, Misset B, Renaud B, Goldstein FW, Vaury P, Carlet J: Mortality of nosocomial pneumonia in ventilated patients: influence of diagnostic tools. Am J Respir Crit Care Med. 1996;154:116-23.
131. Torres A, Aznar R, Gatell JM, Jiménez P, González J, Ferrer A, Celis R, Rodriguez-Roisin R: Incidence, risk, and prognosis factors of nosocomial pneumonia in mechanically ventilated patients. Am Rev Respir Dis. 1990;142:523-8.
132. Trouillet JL, Chastre J, Vuagnat A, Joly-Guillou ML, Combaux D, Dombret MC, Gibert C: Ventilator-associated pneumonia caused by potentially drug-resistant bacteria. Am J Respir Crit Care Med. 1998;157(2):531-9.
133. Urli T, Perone G, Acquarolo A, Zappa S, Antonini B, Ciani A: Surveillance of infections acquired in intensive care: usefulness in clinical practice. *J Hosp Infect* 2002, 52:130-5.
134. Valles J, Pobo A, Garcia-Esquirol O, Mariscal D, Real J, Fernández R. Excess ICU mortality attributable to ventilator-associated pneumonia: the role of early vs late onset. *Intensive care medicine*, 2007;33(8):1363-1368.
135. Vanhems P, Bénet T, Voirin N, Januel JM, Lepape A, Allaouchiche B, Argaud L, Chassard D, Guérin C. Early-onset ventilator-associated pneumonia incidence in intensive care units: a surveillance-based study. BMC Infect Dis. 2011;11(1):236.
136. Verhamme KM, De Coster W, De Roo L, De Beenhouwer H, Nollet G, Verbeke J, Demeyer I, Jordens P: Pathogens in early-onset and late-onset intensive care unit–acquired pneumonia. Infection Control Hospital Epidemiol. 2007;28(4):389-397.
137. Violan JS, Sanchez-Ramirez C, Mujica AP, Cendrero JC, Fernandez JA, de Castro FR: Impact of nosocomial pneumonia on the outcome of mechanically-ventilated patients. Crit Care (Lond). 1998;2:19-23.
138. Warren DK, Zack JE, Elward AM, Cox MJ, Fraser VJ. Nosocomial primary bloodstream infections in intensive care unit patients in a nonteaching community medical center: a 21-month prospective study. *Clin Infect Dis.* 2001;33(8):1329-35.
139. Woske HJ, Röding T, Schulz I, Lode H: Ventilator-associated pneumonia in a surgical intensive care unit Epidemiology, etiology and comparison of three bronchoscopic methods for microbiological specimen sampling. Crit Care. 2001;5:167–173.
140. Xie DS, Xiong W, Lai RP, Liu L, Gan XM, Wang XH, Nie SF. Ventilator-associated pneumonia in intensive care units in Hubei Province, China: a multicentre prospective cohort survey. J Hosp Infect 2011;78(4): 284-288
141. Xie J, Li S, Xue M, Yang C, Huang Y, Chihade DB, Liu L, Yang Y, Qiu H. Early-and Late-Onset Bloodstream Infections in the Intensive Care Unit: A Retrospective 5-Year Study of Patients at a University Hospital in China. *J Infect Dis.* 16;221(Supplement_2):S184-92.
142. Zahar JR, Nguile-Makao M, Français A, Schwebel C, Garrouste-Orgeas M, Goldgran-Toledano D, Azoulay E, Thuong M, Jamali S, Cohen Y, De Lassence A. Predicting the risk of documented ventilator-associated pneumonia for benchmarking: construction and validation of a score. Crit care med. 2009;37(9):2545-51.
143. Acosta-Escribano J, Fernández-Vivas M, Carmona TG, Caturla-Such J, Garcia-Martinez M, Menendez-Mainer A, Sanchez-Payá J (2010) Gastric versus transpyloric feeding in severe traumatic brain injury: a prospective, randomized trial. Intensive Care Med 36:1532-1539
144. Bonten MJ, Gaillard CA, Van der Geest S, Van Tiel FH, Beysens AJ, Smeets HG, Stobberingh EE: The role of intragastric acidity and stress ulcer prophylaxis on colonization and infection in mechanically ventilated ICU patients. A stratified, randomized, double-blind study of sucralfate versus antacids. Am J Respir Crit Care Med. 1995;152:1825-1834.
145. Cook D, Guyatt G, Marshall J, et al A comparison of sucralfate and ranitidine for the prevention of upper gastrointestinal bleeding in patients requiring mechanical ventilation. Canadian Critical Care Trials Group. N Engl J Med 1998;338:791-797
146. Daumal F, Colpart E, Manoury B, Mariani M, Daumal M. Changing heat and moisture exchangers every 48 hours does not increase the incidence of nosocomial pneumonia. Infection Control & Hospital Epidemiology. 1999;20(5):347-9.
147. Djedaini K, Billiard M, Mier L, Le Bourdelles G, Brun P, Markowicz P, Estagnasie P, Coste F, Boussougant Y, Dreyfuss D: Changing heat and moisture exchangers every 48 hours rather than 24 hours does not affect their efficacy and the incidence of nosocomial pneumonia. Am J Respir Crit Care Med. 1995;152(5):1562-9.
148. Drakulovic MB, Torres A, Bauer TT, Nicolas JM, Nogué S, Ferrer M: Supine body position as a risk factor for nosocomial pneumonia in mechanically ventilated patients: a randomised trial. Lancet. 1999;354(9193):1851-1858
149. Dreyfuss D, Djedaini K, Weber P, Brun P, Lanore JJ, Rahmani J, Coste F: Prospective study of nosocomial pneumonia and of patient and circuit colonization during mechanical ventilation with circuit changes every 48 hours versus no change. Am Rev Respir Dis. 1991;143(4 Pt 1), 738-743.
150. Dreyfuss D, Djedaïni K, Gros I, Mier L, Le Bourdellés G, Cohen Y, Estagnasié P, Coste F, Boussougant Y: Mechanical ventilation with heated humidifiers or heat and moisture exchangers: effects on patient colonization and incidence of nosocomial pneumonia. Am J Respir Crit Care Med. 1995;151:986-92.
151. Driks MR, Craven DE, Celli BR, et al (1987) Nosocomial pneumonia in intubated patients given sucralfate as compared with antacids or histamine type 2 blockers. The role of gastric colonization. N Engl J Med 317:1376-1382
152. Forestier C, Guelon D, Cluytens V, Guillart T, Sirot J, De champs C: Oral probiotic and prevention of *Pseudomonas aeruginosa* infections: a randomized, double-blind, placebo controlled pilot study in intensive care unit patients. *Crit Care* 2008;12:R69.
153. Heyland DK, Cook DJ, Schoenfeld PS, Frietag A, Varon J, Wood G The effect of acidified enteral feeds on gastric colonization in critically ill patients: results of a multicenter randomized trial. Canadian Critical Care Trials Group. Crit Care Med 1999;27:2399-2406
154. Holzapfel L, Chastang C, Demingeon G, Bohe J, Piralla B, Coupry A: A randomized study assessing the systematic search for maxillary sinusitis in nasotracheally mechanically ventilated patients. Influence of nosocomial maxillary sinusitis on the occurrence of ventilator-associated pneumonia. Am J Respir Crit Care Med. 1999;159:695-701
155. Kappstein I, Schulgen G, Friedrich T, Hellinger P, Benzing A, Geiger K, Daschner FD. Incidence of pneumonia in mechanically ventilated patients treated with sucralfate or cimetidine as prophylaxis for stress bleeding: bacterial colonization of the stomach. Am J Med. 1991;91(2):S125-31
156. Kirschenbaum L, Azzi E, Sfeir T, et al. Effect of continuous lateral rotational therapy on the prevalence of ventilator-associated pneumonia in patients requiring long-term ventilatory care *Crit Care Med* 2002;30:1983-6.
157. Kirton OC, DeHaven B, Morgan J, et al. A prospective, randomized comparison of an in-line heat moisture exchange filter and heated wire humidifiers: rates of ventilator-associated early-onset (community-acquired) or late-onset (hospital-acquired) pneumonia and incidence of endotracheal tube occlusion. *Chest* 1997;112:1055-9.
158. Knight DJ, Gardiner D, Banks A, Snape SE, Weston VC, Bengmark S, Girling KJ: Effect of synbiotic therapy on the incidence of ventilator associated pneumonia in critically ill patients: a randomised, double-blind, placebo-controlled trial. Intensive Care Med. 2009;35:854-861.
159. Kollef MH, Afessa B, Anzueto A, Veremakis C, Kerr KM, Margolis BD, Schinner R: Silver-coated endotracheal tubes and incidence of ventilator-associated pneumonia: the NASCENT randomized trial. JAMA. 2008;300(7):805-813
160. Lacherade JC, Auburtin M, Cerf C, Van de Louw A, Soufir L, Rebufat Y, Rezaiguia S, Ricard JD, Lellouche F, Brun-Buisson C, Brochard L: Impact of humidification systems on ventilator-associated pneumonia: a randomized multicenter trial. Am J Respir Crit Care Med. 2005;172:1276-1282
161. Lacherade JC, De Jonghe B, Guezennec P, Debbat K, Hayon J, Monsel A, Bastuji-Garin S: Intermittent subglottic secretion drainage and ventilator-associated pneumonia A multicenter trial. Am J Respir Crit Care Med. 2010;182:910-917.
162. Lorente L, Lecuona M, Málaga J, Revert C, Mora ML, Sierra A: Bacterial filters in respiratory circuits: an unnecessary cost? Crit Care Med 2003;31:2126-2130
163. Lorente L, Lecuona M, Galván R, Ramos MJ, Mora ML, Sierra A: Periodically changing ventilator circuits is not necessary to prevent ventilator-associated pneumonia when a heat and moisture exchanger is used. Infect Control Hosp Epidemiol. 2004;25:1077-1082
164. Lorente L, Lecuona M, Martín MM, García C, Mora ML, Sierra A: Ventilator-associated pneumonia using a closed versus an open tracheal suction system. Crit Care Med. 2005;33:115-119
165. Lorente L, Lecuona M, Jiménez A, Mora ML, Sierra A: Tracheal suction by closed system without daily change versus open system. Intensive Care Med. 2006;32:538-44.
166. Lorente L, Lecuona M, Jimenez A, Mora ML, Sierra A: Ventilator-associated pneumonia using a heated humidifier or a heat and moisture exchanger: a randomized controlled trial [ISRCTN88724583]. Crit Care 2006;10:R116
167. Lorente L, Lecuona M, Jimenez A, Mora ML, Sierra: Influence of an endotracheal tube with polyurethane cuff and subglottic secretion drainage on pneumonia. Am J Respir Crit Care Med. 2007;176:1079-1083
168. Lorente L, Lecuona M, Jiménez A, Lorenzo L, Roca I, Cabrera J, Llanos C, Mora ML: Continuous endotracheal tube cuff pressure control system protects against ventilator-associated pneumonia. Crit Care. 2014;18(2):1.
169. Manzano F, Fernandez-Mondejar E, Colmenero M, Poyatos ME, Rivera R, Machado J, Catalan I, Artigas A: Positive-end expiratory pressure reduces incidence of ventilator-associated pneumonia in nonhypoxemic patients. Crit Care Med: 2008;36(8):2225-31.
170. Martin C, Perrin G, Gevaudan MJ, Saux P, Gouin F. Heat and moisture exchangers and vaporizing humidifiers in the intensive care unit. Chest. 1990;97(1):144-9.
171. Morrow LE, Kollef MH, Casale TB: Probiotic prophylaxis of ventilator-associated pneumonia: a blinded, randomized, controlled trial. Am J Respir Crit Care Med. 2010;182:1058-1064
172. Nseir S, Zerimech F, Fournier C, Lubret R, Ramon P, Durocher A, Balduyck M: Continuous control of tracheal cuff pressure and microaspiration of gastric contents in critically ill patients. Am J Respir Crit Care Med. 2011;184(9):1041-7.
173. Pickworth KK, Falcone RE, Hoogeboom JE, et al Occurrence of nosocomial pneumonia in mechanically ventilated trauma patients: a comparison of sucralfate and ranitidine. Crit Care Med 1993;21:1856-1862
174. Pneumatikos I, Konstantonis D, Tsagaris I, Theodorou V, Vretzakis G, Danielides V, Bouros D: Prevention of nosocomial maxillary sinusitis in the ICU: the effects of topically applied alpha-adrenergic agonists and corticosteroids. Intensive Care Med. 2006;32:532-537
175. Prod'hom G, Leuenberger P, Koerfer J, Blum A, Chiolero R, Schaller MD, Perret C, Spinnler O, Blondel J, Siegrist H, Saghafi L: Nosocomial pneumonia in mechanically ventilated patients receiving antacid, ranitidine, or sucralfate as prophylaxis for stress ulcer. A randomized controlled trial. Ann Intern Med. 1994;120:653-62.
176. Reignier J, Mercier E, Le Gouge A, Boulain T, Desachy A, Bellec F, Lascarrou JB: Effect of Not Monitoring Residual Gastric Volume on Risk of Ventilator-Associated Pneumonia in Adults Receiving Mechanical Ventilation and Early Enteral Feeding. A Randomized Controlled Trial. *JAMA* 2013, 309;249-256.
177. Rumbak MJ, Truncale T, Newton MN, Adams B, Hazard P. A Prospective, Randomized Study Comparing Early Versus Delayed Percutaneous Tracheostomy In Critically Ill Medical Patients Requiring Prolonged Mechanical Ventilation. Chest. 2000;118(4):97S-8S.
178. Ryan P, Dawson J, Teres D, Celoria G, Navab F: Nosocomial pneumonia during stress ulcer prophylaxis with cimetidine and sucralfate. Arch Surg. 1993;128(12):1353-7.
179. Smulders K, van der Hoeven H, Weers-Pothoff I, Vandenbroucke-Grauls C A randomized clinical trial of intermittent subglottic secretion drainage in patients receiving mechanical ventilation. Chest 2002;121:858-862
180. Staudinger T, Bojic A, Holzinger U, Meyer B, Rohwer M, Mallner F, Locker GJ Continuous lateral rotation therapy to prevent ventilator-associated pneumonia Crit Care Med 2010;38(2):486-490
181. Thomachot L, Viviand X, Arnaud S, Boisson C, Martin CD: Comparing two heat and moisture exchangers, one hydrophobic and one hygroscopic, on humidifying efficacy and the rate of nosocomial pneumonia. Chest. 1998;114:1383-1389
182. Thomachot L, Leone M, Razzouk K, Antonini F, Vialet R, Martin C: Do the components of heat and moisture exchanger filters affect humidifying efficacy and the incidence of nosocomial pneumonia? Crit Care Med. 1999;27:923–928
183. Thomachot L, Leone M, Razzouk K, Antonini F, Vialet R, Martin C: Randomized Clinical Trial of Extended Use of a Hydrophobic Condenser Humidifier: 1 vs 7 Days. Crit Care Med. 2002;30:232-7
184. Valencia M, Ferrer M, Farre R, Navajas D, Badia JR, Nicolas JM, Torres A: Automatic control of tracheal tube cuff pressure in ventilated patients in semirecumbent position: a randomized trial. Crit Care Med. 2007;35: 1543-9.
185. Walaszek M, Gniadek A, Kolpa M, Wolak Z, Kosiarska A. The effect of subglottic secretion drainage on the incidence of ventilator associated pneumonia. *Biomed Pap Med Fac Univ Palacky Olomouc Czech Repub*. 2017;161(4):374-80.
186. Zeng J, Wang CT, Zhang FS, Qi F, Wang SF, Ma S, Wu TJ, Tian H, Tian ZT, Zhang SL, Qu Y. Effect of probiotics on the incidence of ventilator-associated pneumonia in critically ill patients: a randomized controlled multicenter trial. Intens care med. 2016;42(6):1018-28.
187. Bellissimo-Rodrigues WT, Menegueti MG, Gaspar GG, Nicolini EA, Auxiliadora-Martins M, Basile-Filho A, Bellissimo-Rodrigues F. Effectiveness of a Dental Care Intervention in the Prevention of Lower Respiratory Tract Nosocomial Infections among Intensive Care Patients: A Randomized Clinical Trial. Infect Control Hosp Epidemiol 2014, 35:1342-1348
188. Bleasdale SC, Trick WE, Gonzalez IM, Lyles RD, Hayden MK, Weinstein RA. Effectiveness of chlorhexidine bathing to reduce catheter-associated bloodstream infections in medical intensive care unit patients. Arch intern med. 2007;167(19):2073-9.
189. Ćabov T, Macan D, Husedžinović I, Škrlin-Šubić J, Bošnjak D, Šestan-Crnek S, Perić B, Kovač Z, Golubović V. The impact of oral health and 0.2% chlorhexidine oral gel on the prevalence of nosocomial infections in surgical intensive-care patients: a randomized placebo-controlled study. Einfluss von Mundgesundheit und von 0, 2% Chlorhexidin-Gel auf die Entwicklung von nosokomialen Infektionen bei Patienten auf einer chirurgischen Intensivstation. Wiener klinische Wochenschrift. 2010;122(13-14):397-404.
190. Caruso P, Denari S, Ruiz SA, Demarzo SE, Deheinzelin D. Saline instillation before tracheal suctioning decreases the incidence of ventilator-associated pneumonia. Crit Care Med 2009, 37:32-38.
191. Climo MW, Yokoe DS, Warren DK et al. Effect of daily chlorhexidine bathing on hospital-acquired infection. *N Engl J Med* 2013; 368: 533–542.
192. Fourrier FE, Cau-Pottier H, Boutigny M, Roussel-Delvallez M, Jourdain, Chopin C: Effects of dental plaque antiseptic decontamination on bacterial colonization and nosocomial infections in critically ill patients. Intensive Care Med. 2000;26:1239-1247
193. Fourrier F, Dubois D, Pronnier P, Herbecq P, Leroy O, Desmettre T, Roussel-Delvallez M: Effect of gingival and dental plaque antiseptic decontamination on nosocomial infections acquired in the intensive care unit a double-blind placebo-controlled multicenter study. Crit Care Med. 2005;33:1728-1735
194. Koeman M, van der Ven AJ, Hak E, et al. Oral decontamination with chlorhexidine reduces the incidence of ventilator-associated pneumonia. Am J Respir Crit Care Med 2006;173:1348-1355
195. Kollef M, Pittet D, Sanchez Garcia M, et al. A randomized double-blind trial of iseganan in prevention of ventilator-associated pneumonia. *Am J Respir Crit Care Med* 2006: 173:91-7.
196. Lorente L, Lecuona M, Jiménez A, Palmero S, Pastor E, Lafuente N, Ramos MJ, Mora ML, Sierra A: Ventilator-associated pneumonia with or without toothbrushing a randomized controlled trial. *Eur J Clin Microbiol Infect Dis.* 2012;31:1-9
197. Milstone AM, Elward A, Song X, Zerr DM, Orscheln R, Speck K, Obeng D, Reich NG, Coffin SE, Perl TM, Pediatric SCRUB Trial Study Group. Daily chlorhexidine bathing to reduce bacteraemia in critically ill children: a multicentre, cluster-randomised, crossover trial. *The Lancet*. 2013;381(9872):1099-106.
198. Mori H, Hirasawa H, Oda S, Shiga H, Matsuda K, Nakamura M: Oral care reduces incidence of ventilator-associated pneumonia in ICU populations. Intensive Care Med, 2006, 32(2), 230-236.
199. Noto MJ, Domenico HJ, Byrne DW, Talbot T, Rice TW, Bernard GR, Wheeler AP. Chlorhexidine bathing and health care–associated infections: a randomized clinical trial. *JAMA*. 2015;313(4):369-78.
200. Seguin P, Tanguy M , Laviolle B, Tirel O, Malledant Y: Effect of oropharyngeal decontamination by povidone-iodine on ventilator-associated pneumonia in patients with head trauma. *Crit Care Med* 2006, 34:1514-1519.
201. Seguin P, Laviolle B, Dahyot-Fizelier C, Dumont R, Veber B, Gergaud S, Asehnoune K, Mimoz O, Donnio PY, Bellissant E, Malledant Y. Effect of oropharyngeal povidone-iodine preventive oral care on ventilator-associated pneumonia in severely brain-injured or cerebral hemorrhage patients: a multicenter, randomized controlled trial. Crit care med. 2014;42(1):1-8.
202. Swan JT, Ashton CM, Bui LN, Pham VP, Shirkey BA, Blackshear JE, Bersamin JB, Pomer RM, Johnson ML, Magtoto AD, Butler MO. Effect of chlorhexidine bathing every other day on prevention of hospital-acquired infections in the surgical ICU: a single-center, randomized controlled trial. Crit Care Med. 2016;44(10):1822-32.
203. Wittekamp BH, Plantinga NL, Cooper BS, Lopez-Contreras J, Coll P, Mancebo J, Wise MP, Morgan MP, Depuydt P, Boelens J, Dugernier T. Decontamination strategies and bloodstream infections with antibiotic-resistant microorganisms in ventilated patients: a randomized clinical trial. *JAMA*. 2018.
204. Bergmans DC, Bonten MJ, Gaillard CA, et al Prevention of ventilator-associated pneumonia by oral decontamination: a prospective, randomized, double-blind, placebo-controlled study. Am J Respir Crit Care Med 2001;164:382-388
205. Bonten MJ, Gaillard CA, Johanson Jr WG, Van Tiel FH, Smeets HG, Van Der Geest S, Stobberingh EE. Colonization in patients receiving and not receiving topical antimicrobial prophylaxis. *Am J Respir Crit Care Med* 1994;150(5):1332-1340.
206. Camus C, Salomon S, Bouchigny C, Gacouin A, Lavoué S, Donnio PY, Javaudin L, Chapplain JM, Uhel F, Le Tulzo Y, Bellissant E. Short-term decline in all-cause acquired infections with the routine use of a decontamination regimen combining topical polymyxin, tobramycin, and amphotericin B with mupirocin and chlorhexidine in the ICU: a single-center experience. *Crit Care Med.* 2014;42(5):1121-30.
207. De la court Jara R, Sigaloff KC, Groot T, van der Spoel JI, Schade RP. Reducing the dosing frequency of selective digestive tract decontamination to three times daily provides effective decontamination of Gram-negative bacteria. *Eur J Clin Microbiol Infect Dis*. 2021 1:1-8.
208. de Smet AMGA, Kluytmans JAJW, Cooper BS, Mascini EM, Benus RFJ, van der Werf TS, van der Hoeven JG, Pickkers P, Bogaers-Hofman D, van der Meer NJ, Bernards AT, Kuijper EJ, Joore JC, Leverstein-van Hall MA, Bindels AJ, Jansz AR, Wesselink RM, de Jongh BM, Dennesen PJ, van Asselt GJ, te Velde LF, Frenay IH, Kaasjager K, Bosch FH, van Iterson M, Thijsen SF, Kluge GH, Pauw W, de Vries JW, Kaan JA, Arends JP, Aarts LP, Sturm PD, Harinck HI, Voss A, Uijtendaal EV, Blok HE, Thieme Groen ES, Pouw ME, Kalkman CJ, Bonten MJ: Decontamination of the digestive tract and oropharynx in ICU patients. *N Engl J Med* 2009, 360:20–31.
209. Frencken JF, Wittekamp BH, Plantinga NL, Spitoni C, van de Groep K, Cremer OL, Bonten MJ. Associations Between Enteral Colonization With Gram-Negative Bacteria and Intensive Care Unit–Acquired Infections and Colonization of the Respiratory Tract. *Clin Infect Dis*. 2017;66(4):497-503.
210. Godard J, Guillaume C, Reverdy ME, Bachmann P, Bui-Xuan B, Nageotte A, Motin J: Intestinal decontamination in a polyvalent ICU. A double-blind study. *Intensive Care Med* 1990, 16:307-311.
211. Gorensek MJ, Carey WD, Washington 2nd JA, Vogt DP, Broughan TA, Westveer MK. Selective bowel decontamination with quinolones and nystatin reduces gram-negative and fungal infections in orthotopic liver transplant recipients. Cleveland Clin J Med. 1993;60(2):139-44.
212. Hartenauer UB, Thülig B, Lawin P, Fegeler W. Infection surveillance and selective decontamination of the digestive tract (SDD) in critically ill patients—results of a controlled study. *Infection.* 1990;18(1):S22-30.
213. Hjortrup A, Rasmussen A, Hansen BA, Hoiby N, Heslet L, Moesgaard F, Kirkegaard P (1997) Early bacterial and fungal infections in liver transplantation after oral selective bowel decontamination. *Transplantation proceedings* 29:3106-3110
214. Konrad F, Schwalbe B, Heeg K, et al. [Frequency of colonization and pneumonia and development of resistance in long-term ventilated intensive-care patients subjected to selective decontamination of the digestive tract]. *Anaesthesist* 1989;38:99-109.
215. Landelle C, Boyer VN, Abbas M, Genevois E, Abidi N, Naimo S, Raulais R, Bouchoud L, Boroli F, Terrisse H, Bosson JL. Impact of a multifaceted prevention program on ventilator-associated pneumonia including selective oropharyngeal decontamination. *Intensive Care Med* 2018;44(11):1777-86.
216. Ledingham I, Eastaway A, Mckay I, Alcock S, Mcdonald J, Ramsay G: Triple regimen of selective decontamination of the digestive tract, systemic cefotaxime, and microbiological surveillance for prevention of acquired infection in intensive care. *Lancet* 1988, 1:785-90.
217. Leone M, Bourgoin A, Giuly E, et al. Influence on outcome of ventilator-associated pneumonia in multiple trauma patients with head trauma treated with selected digestive decontamination. *Crit Care Med* 2002; 30:1741-6.
218. Mathieu C, Abbate R, Meresse Z, Hammad E, Duclos G, Antonini F, Cassir N, Schouten J, Zieleskiewicz L, Leone M. Decreased duration of intravenous cephalosporins in intensive care unit patients with selective digestive decontamination: a retrospective before-and-after study. *Eur J Clin Microbiol Infect Dis*. 2020;39(11):2115-20.
219. Nardi G, Di Silvestre A, De Monte A, Massarutti D, Proietti A, Troncon MG, Zussino M: Reduction in gram-positive pneumonia and antibiotic consumption following the use of a SDD protocol including nasal and oral mupirocin. Eur J Emerg Med 2001;8:203-214
220. Ong DS, Bonten MJ, Safdari K, Spitoni C, Frencken JF, Witteveen E, Horn J, Klein Klouwenberg PM, Cremer OL, MARS consortium, de Beer FM. Epidemiology, management, and risk-adjusted mortality of ICU-acquired enterococcal bacteremia. *Clin Infect Dis* 2015;61(9):1413-20.
221. Oostdijk EAN, Kesecioglu J, Schultz MJ, et al. Notice of Retraction and Replacement: Oostdijk et al. Effects of Decontamination of the Oropharynx and Intestinal Tract on Antibiotic Resistance in ICUs: A Randomized Clinical Trial. *JAMA*. 2014;312(14):1429-1437. *JAMA* 2017
222. Oostdijk EA, de Smet AM, Kesecioglu J, Bonten MJ. The role of intestinal colonization with gram-negative bacteria as a source for intensive care unit-acquired bacteremia. *Crit Care. Med.* 2011;39(5):961-6.
223. Rouby JJ, Poete P, de Lassale EM, Nicolas MH, Bodin L, Jarlier V, Korinek AM, Viars P. Prevention of Gram negative noscomial bronchopneumonia by intratracheal colistin in critically ill patients. *Intensive Care Med*. 1994;20(3):187-92.
224. Silvestri L, Bragadin CM, Milanese M, Gregori D, Consales C, Gullo A, Van Saene HK. Are most ICU infections really nosocomial? A prospective observational cohort study in mechanically ventilated patients. *J Hosp Infect.* 1999;42(2):125-33.
225. Steffen R, Reinhartz O, Blumhardt G, Bechstein WO, Raakow R, Langrehr JM, Rossaint R, Slama K, Neuhaus P. Bacterial and fungal colonization and infections using oral selective bowel decontamination in orthotopic liver transplantations. *Transpl Inter.* 1994;7(2):101-8.
226. Stoutenbeek CP, Van Saene HK, Miranda DR, Zandstra DF. The effect of selective decontamination of the digestive tract on colonisation and infection rate in multiple trauma patients. Intens Care Med. 1984;10(4):185-92.
227. Stoutenbeek CP, van Saene HK, Miranda DR, Zandstra DF, Langrehr D; The effect of oropharyngeal decontamination using topical nonabsorbable antibiotics on the incidence of nosocomial respiratory tract infections in multiple trauma patients. J Trauma 1987;27:357-364
228. Vallés J, Peredo R, Burgueño MJ, de Freitas AP, Millán S, Espasa M, Martín-Loeches I, Ferrer R, Suarez D, Artigas A. Efficacy of single-dose antibiotic against early-onset pneumonia in comatose patients who are ventilated. *Chest*. 2013;143(5):1219-25.
229. Veelo DP, Bulut T, Dongelmans DA, et al. The incidence and microbial spectrum of ventilator-associated pneumonia after tracheotomy in a selective decontamination of the digestive tract-setting. *J Infect* 2008; 56:20-6.
230. Winter R, Humphreys H, Pick A, MacGowan AP, Willatts SM, Speller DC: A controlled trial of selective decontamination of the digestive tract in intensive care and its effect on nosocomial infection. J Antimicrob Chemother. 1992;30:73-87
231. Abele-Horn M, Dauber A, Bauernfeind A, Russwurm W, Seyfarth-Metzger I, Gleich P, Ruckdeschel G: Decrease in nosocomial pneumonia in ventilated patients by selective oropharyngeal decontamination (SOD). Intensive Care Med. 1997;23:187-95.
232. Acquarolo A, Urli T, Perone G, Giannotti C, Candiani A, Latronico N. Antibiotic prophylaxis of early onset pneumonia in critically ill comatose patients. A randomized study. Intensive Care Med. 2005; 31(4):510-6.
233. Aerdts SJ, van Dalen R, Clasener HA, Festen J, van Lier HJ, Vollaard EJ: Antibiotic prophylaxis of respiratory tract infection in mechanically ventilated patients. A prospective, blinded, randomized trial of the effect of a novel regimen. Chest. 1991;100:783-791
234. Bion JF, Badger I, Crosby HA, Hutchings P, Kong KL, Baker J, Hutton P, McMaster P, Buckels JA, Elliott TSJ: Selective decontamination of the digestive tract reduces gram-negative pulmonary colonization but not systemic endotoxemia in patients undergoing elective liver transplantation. Crit Care Med. 1994;22:40-49
235. Blair P, Rowlands BJ, Lowry K, Webb H, Armstrong P, Smilie J Selective decontamination of the digestive tract: a stratified, randomized, prospective study in a mixed intensive care unit. Surgery 1991;110:303-309
236. Blaise M, Pateron D, Trinchet JC, Levacher S, Beaugrand M, Pourriat JL. Systemic antibiotic therapy prevents bacterial infection in cirrhotic patients with gastrointestinal hemorrhage. Hepatology. 1994;20(1):34-8.
237. Bouza E, Granda MJ, Hortal J, Barrio JM, Cercenado E, Muñoz P. Pre-emptive broad-spectrum treatment for ventilator-associated pneumonia in high-risk patients. *Intensive Care Med* 2013;39(9):1547-55.
238. Cerra FB, Maddaus MA, Dunn DL, Wells CL, Konstantinides NN, Lehmann SL, Mann HJ. Selective gut decontamination reduces nosocomial infections and length of stay but not mortality or organ failure in surgical intensive care unit patients. Arch Surg 1992;127:163-167.
239. Cockerill FR, 3rd, Muller SR, Anhalt JP, et al. Prevention of infection in critically ill patients by selective decontamination of the digestive tract. *Ann Intern Med* 1992;117:545-53.
240. de La Cal MA, Cerdá E, Garcıa-Hierro P, Van Saene HK. G ómez-Santos D, Negro E & Lorente JA. Survival benefit in critically ill burned patients receiving selective decontamination of the digestive tract: a randomized, placebocontrolled, double-blind trial. Ann Surg. 2005;241:424-30.
241. Ferrer M, Torres A, Gonzalez J, Puig de la Bellacasa J, el-Ebiary M, Roca M, Gatell JM, Rodriguez-Roisin R: Utility of selective digestive decontamination in mechanically ventilated patients. Ann Intern Med. 1994;120:389-395
242. Flaherty J, Nathan C, Kabins SA, Weinstein RA. Pilot trial of selective decontamination for prevention of bacterial infection in an intensive care unit. J Infect Dis 1990;162:1393-1397.
243. Garbino J, Pichard C, Pichna P, Pittet D, Lew D, Romand J (2004) Impact of enteral versus parenteral nutrition on the incidence of fungal infections: a retrospective study in ICU patients on mechanical ventilation with selective digestive decontamination. Clinical Nutrition 2004;23;705-710.
244. Gaussorgues P, Salord M, Sirodot S, Tigaud S, Cagnin S, Gerard M, Robert D. Efficiency of selective decontamination of the digestive tract on the occurrence of nosocomial bacteremia in patients on mechanical ventilation receiving betamimetic therapy. Réan Soins Intens Méd Urg 1991;7:169-174.
245. Hellinger WC, Yao JD, Alvarez S, Blair JE, Cawley JJ, Paya CV, O’Brien PC. A randomized, prospective, double-blinded evaluation of selective bowel decontamination in liver transplantation. Transplantation 2002;73:1904-9.
246. Jacobs S, Foweraker JE, Roberts SE: Effectiveness of selective decontamination of the digestive tract (SDD) in an ICU with a policy encouraging a low gastric pH. Clin Intensive Med. 1992;3:52-58
247. Kerver AJ, Rommes JH, Mevissen-Verhage EA, et al Prevention of colonization and infection in critically ill patients: a prospective randomized study. Crit Care Med 1988;16:1087-1093.
248. Korinek AM, Laisne MJ, Nicolas MH, Raskine L, Deroin V, Sanson-lepors MJ: Selective decontamination of the digestive tract in neurosurgical intensive care unit patients: a double-blind, randomized, placebo-controlled study. Crit Care Med. 1993;21:1466-73.
249. Laggner AN, Tryba M, Georgopoulos A, Lenz K, Grimm G, Graninger W, Schneeweiss B, Druml W: Oropharyngeal decontamination with gentamicin for long-term ventilated patients on stress ulcer prophylaxis with sucralfate? *Wien Klin Wochenschr* 1994, 106:15-19.
250. Palomar M, Alvarez-Lerma F, Jorda R, Bermejo B, Catalan Study Group of Nosocomial Pneumonia Prevention: Prevention of nosocomial infection in mechanically ventilated patients: selective digestive decontamination versus sucralfate. Clin Intens Care. 1997;8:228-235
251. Pneumatikos I, Koulouras V, Nathanail C, Goe D, Nakos G: Selective decontamination of subglottic area in mechanically ventilated patients with multiple trauma. *Intensive Care Med*. 2002;28:432-437
252. Quinio B, Albanese J, Bues-Charbit M, Viviand X, Martin C; Selective decontamination of the digestive tract in multiple trauma patients. A prospective double-blind, randomized, placebo-controlled study. Chest 1996;109:765-772
253. Rocha LA, Martin MJ, Pita S, Paz J, Seco C, Margusino L, Villanueva R, Duran MT: Prevention of nosocomial infection in critically ill patients by selective decontamination of the digestive tract. A randomized, double blind, placebo-controlled study. Intensive Care Med. 1992;18:398-404
254. Rodríguez-Roldán JM, Altuna-Cuesta A, López A, Carrillo A, Garcia J, León J, Martínez-Pellús AJ: Prevention of nosocomial lung infection in ventilated patients: use of an antimicrobial pharyngeal nonabsorbable paste. Crit Care Med. 1990;18:1239-42
255. Rolando N, Gimson A, Wade J, Philpott‐Howard J, Casewell M, Williams R: Prospective controlled trial of selective parenteral and enteral antimicrobial regimen in fulminant liver failure. Hepatol. 1993;17:196-201
256. Rolando N, Wade JJ, Stangou A, Gimson AE, Wendon J, Philpott‐Howard J, Williams R. Prospective study comparing the efficacy of prophylactic parenteral antimicrobials, with or without enteral decontamination, in patients with acute liver failure. Liver transplantation and surgery. 1996; 2:8-13
257. Sanchez-Garcia M, Cambronero JA, Lopez-Diaz J, et al. Effectiveness and cost of selective decontamination of the digestive tract in critically ill intubated patients. A randomized, double-blind, placebo-controlled multicenter trial. *Am Rev Respir Dis* 1998; 158:908-16.
258. Sirvent JM, Torres A, El-Ebiary M, Castro P, de Batlle J, Bonet A. Protective effect of intravenously administered cefuroxime against nosocomial pneumonia in patients with structural coma. *Am J Respir Crit Care Med* 1997;155:1729-1734
259. Smith SD, Jackson RJ, Hannakan CJ, Wadowsky RM, Tzakis AG, Rowe MI; Selective decontamination in pediatric liver transplants. Transplantation 1993;55:1306-1308
260. Stoutenbeek CP, van Saene HKF, Little RA, Whitehead A: The effect of selective decontamination of the digestive tract on mortality in multiple trauma patients: a multicenter randomized controlled trial. Intensive Care Med. 2007;33:261-270
261. Ulrich C, Harinck-deWeerd JE, Bakker NC, et al Selective decontamination of the digestive tract with norfloxacin in the prevention of ICU-acquired infections: A prospective randomized study. Intensive Care Med 1989;15:424-431.
262. Unertl K, Ruckdeschel G, Selbmann HK, et al; Prevention of colonization and respiratory infections in long-term ventilated patients by local antimicrobial prophylaxis. Intensive Care Med 1987;13:106-113
263. Van Delden C, Köhler T, Brunner-Ferber F, François B, Carlet J, Pechère JC. Azithromycin to prevent Pseudomonas aeruginosa ventilator-associated pneumonia by inhibition of quorum sensing: a randomized controlled trial. Intensive Care Med. 2012 Jul 1;38(7):1118-25.
264. Verwaest C, Verhaegen J, Ferdinande P, Schetz M, Van den Berghe G, Verbist L, Lauwers P: Randomized, controlled trial of selective digestive decontamination in 600 mechanically ventilated patients in a multidisciplinary intensive care unit. Crit Care Med. 1997;25:63-71
265. Wiener J, Itokazu G, Nathan C, Kabins SA, Weinstein RA: A randomized, double-blind, placebo-controlled trial of selective digestive decontamination in a medical-surgical intensive care unit. Clin Infect Dis. 1995;20:861-867
266. Zobel GE, Kuttnig MA, Grubbauer HM, Semmelrock HJ, Thiel WE. Reduction of colonization and infection rate during pediatric intensive care by selective decontamination of the digestive tract. Crit Care Med. 1991;19(10):1242-6
267. Ables AZ, Blumer NA, Valainis GT, Godenick MT, Kajdasz DK, Palesch YY. Fluconazole Prophylaxis of Severe Candida Infection in Trauma and Postsurgical Patients: A Prospective, Double-Blind, Randomized, Placebo-Controlled Trial. Infect Dis Clin Practice 2000;9(4):169-175.
268. Eggimann P, Francioli P, Bille J, Schneider R, Wu MM, Chapuis G, Chiolero R, Pannatier A, Schilling J, Geroulanos S, et al (1999) Fluconazole prophylaxis prevents intra-abdominal candidiasis in high-risk surgical patients. Crit Care Med 27:1066–1072.
269. Garbino J, Lew DP, Romand JA, Hugonnet S, Auckenthaler R, Pittet D: Prevention of severe Candida infections in nonneutropenic, high-risk, critically ill patients: a randomized, double-blind, placebo-controlled trial in patients treated by selective digestive decontamination. Intensive Care Med 2002;28:1708-1717
270. Giglio M, Caggiano G, Dalfino L, Brienza N, Alicino I, Sgobio A, Favale A, Puntillo F. Oral nystatin prophylaxis in surgical/trauma ICU patients: a randomised clinical trial. Crit Care 2012;16(2):R57.
271. Jacobs S, Price Evans DA, Tariq M, Al Omar NF (2003) Fluconazole improves survival in septic shock: a randomized double-blind prospective study. Crit Care Med 31:1938–1946.
272. Lumbreras C, Cuervas-Mons V, Jara P, Del Palacio A, Turrion VS, Barrios C, Paya C V (1996) Randomized trial of fluconazole versus nystatin for the prophylaxis of *Candida* infection following liver transplantation. J Infect Dis 174(3); 583-588.
273. Normand S, François B, Dardé ML, Bouteille B, Bonnivard M, Preux PM, Gastinne H, Vignon P. Oral nystatin prophylaxis of *Candida* spp. colonization in ventilated critically ill patients. Intensive Care Med 2005;31:1508–1513.
274. Ostrosky-Zeichner L, Shoham S, Vazquez J, Reboli A, Betts R, Barron MA, Pappas P G. MSG-01: a randomized, double-blind, placebo-controlled trial of caspofungin prophylaxis followed by pre-emptive therapy for invasive candidiasis in high-risk adults in the critical care setting. Clin Infect Dis 2014;58(9):1219-1226.
275. Parizkova R, Cerny V, Dostal P, Truhlar A (2000) The effect of prophylactic fluconazole administration on fungal infection in critically ill patients. Anesteziologie a neodkladna pece 11:271-5.
276. Pelz RK, Hendrix CW, Swoboda SM, Diener-West M, Merz WG, Hammond J, Lipsett PA. Double-blind placebo-controlled trial of fluconazole to prevent candidal infections in critically ill surgical patients. Annal Surg. 2001;233(4):542.
277. Piarroux R, Grenouillet F, Balvay P, Tran V, Blasco G, Millon L, Boillot A Assessment of pre-emptive treatment to prevent severe candidiasis in critically ill surgical patients. Crit Care Med 2004;32(12):2443-2449.
278. Savino JA, Agarwal N, Wry P, Policastro A, Cerabona T, Austria L. Routine prophylactic antifungal agents (clotrimazole, ketoconazole, and nystatin) in nontransplant nonburned critically ill surgical and trauma patients. J Trauma 1994;36:20–26.
279. Schuster MG, Edwards JE, Sobel JD, Darouiche RO, Karchmer AW, Hadley S, Rex JH (2008) Empirical fluconazole versus placebo for intensive care unit patients: a randomized trial. Ann Intern Med 149(2):83-90.
280. Verhaegen J: Randomized study of selective digestive decontamination on colonization and prevention of infection in mechanically ventilated patients in the ICU. 1992. Doctor in Medical Sciences – thesis, University Hospital, Leuven, Belgium.
281. Klompas M, Speck K, Howell MD, *et al*. Reappraisal of routine oral care with chlorhexidine gluconate for patients receiving mechanical ventilation: systematic review and meta-analysis. *JAMA Intern Med.* 2014; **174**: 751-61.
282. Zhao T, Wu X, Zhang Q, Li C, Worthington HV, Hua F. Oral hygiene care for critically ill patients to prevent ventilator‐associated pneumonia. *Cochrane Database Syst Rev*. 2020(12).
283. Silvestri L, Weir I, Gregori D, Taylor D, Van Saene J, Van Saene H. Effectiveness of oral chlorhexidine on nosocomial pneumonia, causative microorganisms and mortality in critically ill patients: a systematic review and meta-analysis. Minerva Anestesiol. 2014;80(7):805-20.
284. Silvestri L, Weir WI, Gregori D, Taylor N, Zandstra DF, van Saene JJ, van Saene HK. Impact of oral chlorhexidine on bloodstream infection in critically ill patients: systematic review and meta-analysis of randomized controlled trials. J Cardiothoracic Vasc Anesth. 2017;31(6):2236-44.
285. Hurley JC: Prophylaxis with enteral antibiotics in ventilated patients: Selective decontamination or selective cross-infection? *Antimicrob Agents Chemother* 1995; **39**: 941–7.
286. Minozzi S, Pifferi S, Brazzi L, Pecoraro V, Montrucchio G, D'Amico R. Topical antibiotic prophylaxis to reduce respiratory tract infections and mortality in adults receiving mechanical ventilation. Cochrane Database Syst Rev 2021, Issue 1. Art. No.: CD000022. DOI: 10.1002/14651858.CD000022.pub4.
287. Silvestri L, Van Saene HK, Milanese M, Gregori D, Gullo A. Selective decontamination of the digestive tract reduces bacterial bloodstream infection and mortality in critically ill patients. Systematic review of randomized, controlled trials. J Hosp Infect. 2007;65(3):187-203.
288. van Till JO, van Ruler O, Lamme B, Weber RJ, Reitsma JB, Boermeester MA. Single-drug therapy or selective decontamination of the digestive tract as antifungal prophylaxis in critically ill patients: a systematic review. Critical Care. 2007;11(6):1-6.
289. Silvestri L, Van Saene HK, Milanese M, Gregori D. Impact of selective decontamination of the digestive tract on fungal carriage and infection: systematic review of randomized controlled trials. *Intensive Care Med* 2005, 31:898-910.
290. Cortegiani A, Russotto V, Maggiore A, Attanasio M, Naro AR, Raineri SM, Giarratano A. Antifungal agents for preventing fungal infections in non‐neutropenic critically ill patients. Cochrane Database of Systematic Reviews. 2016(1).
291. Playford EG, Webster AC, Sorrell TC, Craig JC. Antifungal agents for preventing fungal infections in non-neutropenic critically ill and surgical patients: systematic review and meta-analysis of randomized clinical trials. *J Antimicrob Chemother* 2006 ;57(4):628-38.

*Figure s1a & b* Scatter plots (logit scale) of Pseudomonas VAP incidence (top) and Pseudomonas bacteremia (bottom) in control (_C) and intervention (_I) component groups of various methods of infection prevention in the ICU (all studies). The benchmark incidence in each plot is the summary mean derived from the observation studies (central vertical line). non-D is non-decontamination and SAF is single drug anti-fungal

*Figure s2a & b* Scatter plots (logit scale) of RT Candida (the count of candida amongst patient with VAP) incidence (top) and Candidemia (bottom) in component control (_C) and intervention (_I) groups of various methods of infection prevention in the ICU (all studies). The benchmark incidence in each plot is the summary mean derived from the observation studies (central vertical line). The groups wide presence of candidemia risk factors (CRF) is identified by solid symbols versus not (open). non-D is non-decontamination and SAF is single drug anti-fungal

*Figure s3* Effect size *VAP Pseudomonas*

*Figure s4* Effect size *Pseudomonas bacteremia*

*[Note; the Pseudomonas counts in the study of Oostdijk 2011 [222] come from the study by De Smet 2009 [208] and are taken as listed in Table 2 of Oostdijk 2011 [222] even though this Table appears to be mislabelled in comparison to the text and table 1 of Oostdijk 2011 [222] and in relation to the data in De Smet 2009 [208]].*

*Figure s5* Effect size *RT candida* (the count of candida amongst patient with VAP)

*Figure s6* Effect size *Candidemia*

*Figure s7. GSEM model C*

*Figure s8. GSEM model B*
